# Supplementary material for: Brain connectivity-guided, Optimised theta burst transcranial magnetic stimulation to improve Central Pain Modulation in knee Osteoarthritis Pain (BoostCPM): protocol of a pilot randomised clinical trial in a secondary care setting in the UK
Source: BMJ Open. 2023 Oct 16;13(10):e073378. doi: 10.1136/bmjopen-2023-073378 (PMC10582853; doi:10.1136/bmjopen-2023-073378)
Supplement: Author's manuscript [file bmjopen-2023-073378.draft_revisions.pdf]

BMJ Open is committed to open peer review. As part of this commitment we make the peer review history of every article we publish publicly available.

When an article is published we post the peer reviewers' comments and the authors' responses online. We also post the versions of the paper that were used during peer review. These are the versions that the peer review comments apply to.

The versions of the paper that follow are the versions that were submitted during the peer review process. They are not the versions of record or the final published versions. They should not be cited or distributed as the published version of this manuscript.

BMJ Open is an open access journal and the full, final, typeset and author-corrected version of record of the manuscript is available on our site with no access controls, subscription charges or pay-per-view fees (http://bmjopen.bmj.com).

If you have any questions on BMJ Open's open peer review process please email info.bmjopen@bmj.com

BMJ Open

Brain connectivity-guided, optimised theta burst transcranial magnetic stimulation to improve central pain modulation in knee osteoarthritis pain (BoostCPM): protocol of a pilot randomised clinical trial

Journal:	*BMJ Open*
Manuscript ID	bmjopen-2023-073378
Article Type:	Protocol
Date Submitted by the Author:	03-Mar-2023
Complete List of Authors:	Drabek, Marianne; University of Nottingham,<br>Hodkinson, Duncan; Nottingham NIHR Biomedical Research Centre; University of Nottingham<br>Horvath, Suzanne; Nottingham NIHR Biomedical Research Centre<br>Millar, Bonnie; University of Nottingham Faculty of Medicine and Health Sciences, Academic Rheumatology; NIHR Nottigham Biomedical Research Centre, Academic Rheumatology<br>Pszczolkowski Parraguez, Stefan; University of Nottingham<br>Tench, Christopher; University of Nottingham, Division of Clinical Neuroscience<br>Tanasescu, Radu; University of Nottingham,<br>Lankappa, Sudheer; Nottinghamshire Healthcare NHS Foundation Trust, Adult Mental Health<br>Morriss, Richard; University of Nottingham, Psychiatry<br>Walsh, David; University of Nottingham, Academic Rheumatology<br>Auer, Dorothee; Nottingham NIHR Biomedical Research Centre; University of Nottingham
Keywords:	Chronic Pain, PAIN MANAGEMENT, PSYCHIATRY

SCHOLARONE™
Manuscripts

1

Brain connectivity-guided, **o**ptimised theta burst transcranial magnetic stimulation to improve **c**entral **p**ain **m**odulation in knee **o**steoarthritis **p**ain (BoostCPM): protocol of a pilot randomised clinical trial

Authors

Marianne M. Drabek[1,2,3], Duncan Hodkinson[1,2,3], Suzanne Horvath[1], Bonnie Millar[1,3], Stefan Pszczolkowski[1,2], Christopher Tench[1,2,5], Radu Tanasescu[1,4,5], Sudheer T Lankappa[6], Richard Morriss[1], David A. Walsh[3], Dorothee P. Auer[1,2,3]

Affiliations

[1] Nottingham NIHR Biomedical Research Centre

[2] Sir Peter Mansfield Imaging Centre, School of Medicine, University of Nottingham, Nottingham

[3] Pain Centre Versus Arthritis, University of Nottingham, Nottingham

[4] Department of Neurology, Queen's Medical Centre, Nottingham University Hospital Trust

[5] Academic Clinical Neurology, School of Medicine, University of Nottingham

[6] Psychiatry, Nottinghamshire Healthcare Foundation Trust

Corresponding author

Dorothee P. Auer, Radiological Sciences, Room W/B 1441, Queen's Medical Centre, Nottingham, NG7 2UH, UK. Email: dorothee.auer@nottingham.ac.uk

Word count: 3939

Key words: Transcranial Magnetic Stimulation, chronic pain, refractory pain, osteoarthritis, brain connectivity, dorsolateral prefrontal cortex

For peer review only - http://bmjopen.bmj.com/site/about/guidelines.xhtml

Abstract

Introduction

Chronic pain is a common health problem that is not efficiently managed by standard analgesic treatments. There is evidence that treatment resistance may result from maladaptive brain changes in areas that are fundamental to the perception of pain. Knee osteoarthritis is one of the most prevalent causes of chronic pain and commonly associated with negative affect. Chronic knee osteoarthritis pain is also associated with altered right anterior insula functional connectivity. We posit that reversal of these brain circuit alterations may be critical to alleviate chronic pain and associated negative affect, and that this can be achieved through non-invasive neuromodulation techniques. Despite growing interest in non-invasive neuromodulation for pain relief and proven efficacy in depression, results in chronic pain are mixed with limited high-quality evidence for clinical and mechanistic efficacy. Limitations include patient heterogeneity, imprecision of target selection, and protocols that may deliver pulses at subclinical efficacy.

Methods and Analysis

We hence developed an optimised treatment protocol of connectivity-guided intermittent theta-burst stimulation (iTBS) targeting the left dorso-lateral prefrontal cortex with accelerated delivery on four consecutive days (allowing four days within the same week as protocol variation) with 5 daily treatment sessions that will be piloted in a sham-controlled design in 45 participants with chronic knee pain, reporting negative affect. This pilot study protocol will assess feasibility, tolerability and explore mechanistic efficacy through serial functional/structural MRI and quantitative sensory testing.

Ethics and Dissemination

This pilot trial has been approved by the Ethics Committee Cornwall & Plymouth.

Strengths and limitations of this study

Strengths:

-improved precision to stimulate the prefronto-insular circuit through connectivity-guided neuronavigation

-accelerated iTBS protocol to maximise efficacy and efficiency

-Sham-controlled RCT design

-MRI monitoring of brain networks before and after TMS protocol.

For peer review only - http://bmjopen.bmj.com/site/about/guidelines.xhtml

-CPM assessment before and after iTBS protocol

Limitations:

-pilot study in participants selected for negative effect.

-intense protocol that may not be accommodated and tolerated by all chronic pain patients.

Introduction

Chronic pain is a major burden for society, economy, health systems and individuals. There is a lack of effective analgesic treatments in chronic pain with an urgent need for novel treatments. Maladaptive brain mechanisms are increasingly recognised as key contributors to the development and persistence of pain (1). A feature is the altered communication between brain areas that are involved in the processing of pain signals and the modulation of the associated pain perception, which partially overlap with the observed brain connectivity changes in major depression (2).

These maladaptive functional brain network changes are thought (i) to drive the experience of chronic pain and central pain sensitisation, (ii) to be potentially reversible by non-invasive neuromodulation, and thus support the notion that interventions that normalise the brain's (and in particular fronto-limbic) connectivity will also reverse central pain augmentation or associated affective experience.

The best-established method to non-invasively change brain networks and thus restore normal connectivity patterns is Transcranial Magnetic Stimulation (TMS) which has been approved by the National Institute of Clinical Excellence (NICE) for treatment-resistant depression. Pain and depression are closely related from the perspective of involved brain regions, which may explain the known comorbidity between both conditions. This suggests that TMS could become useful in treating 'refractory' pain (i.e. chronic pain that failed to get better in multiple treatment attempts) as it is effective for treatment-resistant depression. Efficacy might be higher in patients with higher associated affective symptoms including higher levels of reported pain catastrophizing, low mood or anxiety.

There is evidence that invasive neuromodulation is effective in selected indications (3).There is also considerable recent interest in non-invasive brain stimulation (NIBS) trials in chronic pain with mixed results to date. Meta-analyses and systematic reviews have described NIBS as having 'poor or inconclusive' analgesic effects across sites and pain disorders (3-5), "minimally clinically relevant"(5), to "promising"(6) and "probable efficacy" for either motor cortex (M1) or dorsolateral prefrontal cortex (DLPFC) stimulation (7). There are major quality concerns with most studies considered poor

For peer review only - http://bmjopen.bmj.com/site/about/guidelines.xhtml

(3) due to underpowered sample sizes. Moreover, there are uncertainties regarding the most effective brain stimulation site, type of stimulation, target population. Nevertheless, pain relief was frequently found to be >30% (8) which encourages to seek further improvements and address limitations in order to fully assess and exploit the potential that this technology may hold for pain treatment. In particular the DLPFC as target site for TMS is relatively understudied in chronic pain despite well-established efficacy rates seen in treatment-resistant depression with equal results achieved for standard rTMS and shorter duration theta-burst stimulation (9) and the known interplay between negative affect and chronic pain.

In addition, recently accelerated, optimised TMS protocols were reported to be safe and similarly effective to standard rTMS using intermittent theta burst stimulation (iTBS) both at 80% and 120% resting motor threshold [RMT] using a total of 24,000 pulses over two weeks (10) or 32,400 pulses over one week (11). Remarkably better outcomes were reported using an even more intense protocol with a higher cumulative dose of 90,000 iTBS pulses (90% RMT) delivered over 5 consecutive days using individually optimised treatment target (12).

We aim to translate and adapt this promising approach to chronic musculo-skeletal pain, which, if effective, would greatly improve clinical interest in this technique both for cost and practical reasons. Pain is a sensory and affective experience with a complex interplay of negative affect and pain sensitisation at behavioural and neural level. While the underlying mechanisms are not fully understood, it is plausible to assume that participants with higher levels of reported catastrophizing, anxiety and low mood may particularly benefit from prefrontal neuromodulation.

To address the remaining fundamental questions before undertaking clinical analgesic trials, there is a need for sham-controlled piloting of an accelerated and individually targeted iTBS protocol to assess feasibility and tolerability in the target population, namely chronic knee pain with reported negative affect, and to investigate the variance of mechanistic outcomes using advanced quantitative sensory testing and advanced neuroimaging.

To this end, a parallel, sham-controlled pilot study will assess feasibility, tolerability and explore mechanistic outcome markers of a four day (over one week) intense, imaging-guided intermittent theta burst stimulation protocol that is individually targeted within the left DLPFC to maximise the connectivity with the right anterior insula. Study participants with moderately severe, chronic knee pain and increased negative affect will undergo serial MRI, behavioural and quantitative sensory testing before and after the iTBS/sham intervention and online recording of patient-reported outcomes until week 18 to guide the protocol and sample size for future RCT interventions.

Objectives

For peer review only - http://bmjopen.bmj.com/site/about/guidelines.xhtml

(i) To test the feasibility and tolerability of an optimized accelerated four day (over one week) iTBS protocol with 5 daily sessions (total pulses 36,000 aimed at 80% RMT) using connectivity-guided targeting of the left DLPFC in chronic knee OA patients with moderately severe pain and self-reported negative affect.

(ii) To assess the variance of mechanistic outcome markers comparing the intense iTBS protocol versus sham.

(iii) To explore clinical efficacy metrics, and the feasibility of online recorded patient-reported outcome measures in the iTBS and sham arm.

(iv) To explore the effects of iTBS on negative affect, stress and arousal between iTBS and sham.

(v) To assess feasibility of blinding using sham TMS.

Design and methods

Trial design

This is a single centre sham controlled randomised mechanistic pilot study at the University of Nottingham with a parallel group study design, whereby patients will either receive active iTBS or sham iTBS with random allocation of 2:1.

Hypotheses

Primary mechanistic hypotheses

1. Accelerated, personalised iTBS of the DLPFC modulates *functional activity and connectivity* of brain circuits involved in central pain control in chronic musculoskeletal pain compared with sham controls.
2. Accelerated, personalised iTBS of the DLPFC increases endogenous antinociception in chronic MSK pain compared to sham controls.

Secondary Hypotheses

3. Accelerated, personalised iTBS of the DLPFC reduces pain catastrophizing, anxiety and improves mood in chronic MSK pain compared to sham controls.
4. Accelerated, personalised iTBS of the DLPFC reduces sensitivity to experimental pain at the end of the intervention compared to sham controls.
5. Accelerated, personalised iTBS of the DLPFC reduces physiological arousal and reported stress levels compared to sham controls.

For peer review only - http://bmjopen.bmj.com/site/about/guidelines.xhtml

Study Procedures

Participants will be asked to attend for baseline assessments (visit 1) (ca. 2 hours including MRI, and psycho-physical assessment including brief experimental pain exposure) (see Figure 1). Then participants will undergo an intervention week with four visits (Visit 2-5) lasting half a day. Each visit will consist of five blocks of iTBC or sham with intersession interval of ~50 minutes during which participants will be offered refreshments and food in a comfortable waiting area before getting prepared for the next session. The 6th and final study visit is a repeat of the baseline visit, and will be scheduled within 18 -72 hours of the last treatment session.

Transcranial magnetic stimulation

Transcranial magnetic stimulation (TMS) will be delivered using the Magstim Horizon Performance system with StimGuide Navigated TMS package (The Magstim Co. Ltd). Single-pulse TMS will be applied to the left motor cortex using a handheld figure-of-eight coil (diameter 7cm). Resting motor threshold (RMT) is defined as the minimum stimulator output intensity needed to achieve a minimum motor evoked potential (MEP)-amplitude of 50µV in the completely relaxed APB-muscle in at least 5 out of 10 trials. For all treatments, the intermittent theta burst stimulation (iTBS) protocol will be delivered using a fixed 7cm diameter coil (E-z Cool Coil and support arm) consisting of 3 pulses (aimed at 80% RMT, but will be lowered to avoid participant discomfort) of 50Hz repeated at 200ms (5Hz) intervals with a total 1800 pulses per block/session.

MRI acquisition

Imaging data will be acquired on a GE Premier 3T MR scanner using a 48-channel head coil (GE Healthcare, USA). Structural scans include high resolution T1-weighted (T1w) and T2-weighted (T2w) images for the purpose of image registration. The T1w images acquired using 3D MP-RAGE sequence (voxel size=1mm isotropic, FOV = 256, matrix = 256, 256 sagittal slices, TI = 800 ms, FA = 8°) and T2w images acquired using 3D Cube T2 FLAIR sequence (voxel size=1mm isotropic, FOV = 256, matrix = 256, 256 sagittal slices, TR = 6300ms, TE =121ms, TI =1787ms). The resting-state fMRI data were acquired using whole-brain 2D GE-EPI sequence (TR=1400ms, TE=35ms, flip angle=68°, in-plane FOV = 212×212mm, 57 slices, slice thickness=2mm, voxel size=2mm isotropic, hyperband factor=3, ARC factor=2, 15min total scan time). To measure the b_0 field for correction of EPI distortions, we also

For peer review only - http://bmjopen.bmj.com/site/about/guidelines.xhtml

acquire two SE-EPI images with reversed phase encoding directions. The SE-EPI images have the same geometry, echo spacing, and phase encoding direction parameters as the GE fMRI scans.

Image processing and TMS neuronavigation

Image pre-processing follows the same procedure to that described in Pszczolkowski et al. (13) . In brief, the structural T1-weighted images are brain extracted, bias corrected and segmented into white matter (WM), grey matter (GM) and cerebrospinal fluid (CSF) probability maps which are binarised at a probability of 0.98 and then eroded using a spherical kernel with a radius of 2 voxels to obtain the final tissue labels. The BOLD rsfMRI images undergo EPI distortion correction, motion correction, slice timing correction, smoothing, denoising and high-pass filtering. Finally, the WM and GM tissue labels are transformed into rsfMRI BOLD space and used to regress out the WM and GM time series from the processed rsfMRI BOLD data.

For identification of the iTBS treatment target, a methodology similar to Pszczolkowski et al. (13) is followed. Different here is that the DLPFC searchlight described in Fox et al. (14) is used instead and that the background removal procedure is based on a threshold computed automatically rather than by visual inspection. This threshold is computed as the 98th percentile of the T1-weighted intensities within a mask of background (air) voxels around the outside of the skull in MNI space which is previously transformed into T1-weighted space.

Quantitative sensory testing

Central pain inhibition will be tested using the psychophysical paradigm of conditioned pain modulation (CPM). The test stimulus will be pressure pain threshold (PPT) and the conditioning stimulus ischemic pain caused by a blood pressure cuff placed at the calf of the unaffected leg. The test stimulus (PPTs) will be administered at three body sites (on the affected side), including trapezius muscle, metacarpophalageal joint of the thumb, and the patella.

Conditioned pain modulation is calculated as follows:

CPM (%) = (PPTpost-conditioning – PPTpre-conditioning)/ PPTpre-conditioning X 100.

Pre-conditioning PPTs will be used to calculate SEM and >±2XSEM to determine CPM effects in response TBS treatment.

For peer review only - http://bmjopen.bmj.com/site/about/guidelines.xhtml

For peer review only

Sham, randomisation and blinding

There will be a sham-controlled parallel arm intervention study whereby participants will be blinded to the intervention allocation and participants will be asked whether they can guess the type of intervention. The study team involved in delivering the intervention cannot be blinded, but the data analysis team will be blinded to which intervention was delivered. Sham intervention will be given through the same coil, but pulses will be delivered at 30% stimulator output by orienting the coil perpendicular to the scalp surface with direct contact. This was to ensure an appropriate audible noise is created to reinforce the integrity of the treatment.

Both active and sham will be administered over the left DLPFC using neuronavigation co-ordinates (s. above).

Assessments

Data will be collected for the following

- MRI at visit 1 and at visit 6 (pre- and post-treatment)
- Quantitative sensory testing (QST) at visit 1 and at visit 6 (pre- and post-treatment)

RedCap (https://www.project-redcap.org/) will be used to administer online questionnaires:

Pain characteristics

- a numerical pain VAS (0-10) as online daily pain report
- weekly questionnaires:
  Intermittent and Constant Pain Score (ICOAP) (15), The Central Aspects of Pain in the Knee (CAP-Knee) questionnaire (16)
- at visit 1 &6:
  painDETECT (17), Pain Catastrophizing Scale (PCS)(18), ICOAP (15), General Anxiety Depression scale (GAD-7)(19), Depression Anxiety Stress Scales (DASS-21)(20), Hospital Anxiety and Depression Rating-Scale (HADS)(21), Patient Health Questionnaire (PHQ-9)(22)

Outcome metrics and measures

Outcome metrics are changes between end of the intervention (Visit 6) and baseline (Visit 1) of optimized neuroimaging markers of the DPMS that will be established from independent dataset and pre-defined in the analysis plan and 2) Central pain modulation, derived from QST.

Outcome measures

For peer review only - http://bmjopen.bmj.com/site/about/guidelines.xhtml

Primary:

1. Variance of changes in pre-selected neuroimaging functional markers of the descending pain modulatory system at final vs baseline visit.
2. Variance of change in conditioned pain modulation (pain intensity change with / without conditioned pain stimulus) at final vs baseline visit.
3. Number of participants recruited and retained on the trial.
4. Number of iTBS sessions completed at 80% RMT.

Secondary:

5. Variance of change in reported pain intensity, burden and catastrophizing at final versus baseline study visit (ICOAP, PCS)
6. Change in negative affect (HADS) at final versus baseline study visit.
7. Variance in physiological and psychophysical changes (acute experimental pain) at final vs baseline study visit.
8. Variance of PROMS (daily online recording pain, wellbeing, sleep) over 16 weeks from treatment versus enrolment-baseline.

Recruitment and inclusion criteria

Participants will be recruited via posters, general practitioner surgeries and other NHS services (Joint replacement waiting list), previous participants who consented to be contacted (such as the Muscular Health and Wellbeing cohort)(23) , co-recruited from ongoing studies that allow co-recruitment, or in response to publicly placed posters. Individuals who consented to be contacted about new studies, will be contacted by the study team who originally consented the participant; only if the participant then expresses interest in the new study and agrees for their contact details to be passed on to the new study team, the latter will contact them.

Participants may be initially invited to take part in the study by sending a short format participant information sheet (PIS) form that summarizes the main points of the study for consideration of interest; participants who contact for further information will be sent the final PIS. Alternatively, they may find the short format PIS as poster and contact us. Posters may be put around the Nottingham University Hospital Trust, the Medical School and other University of Nottingham sites, public sites such as public libraries and leisure/community centres.

Posters may be placed on local press, including but not limited to The Nottingham Post and Metro. The same advert may be featured on UK websites related to chronic pain, including but not limited to the Pain Centre Versus Arthritis webpages within http://www.nottingham.ac.uk/paincentre.

For peer review only - http://bmjopen.bmj.com/site/about/guidelines.xhtml

Participants will be reimbursed for travel expenses and provided with free drinks and food during study sessions but will not be compensated for their time.

Inclusion criteria

- 18-75 years of age and able to consent
- Chronic knee pain (minimum 6 months duration) with VAS score >=4/10
- Predominant knee pain
- Reporting low mood, high anxiety or pain catastrophizing with scores above population average in one of three domains (mood, anxiety, Pain Catastrophizing Score)
- Able to accommodate the study visits without disruption to their jobs or other responsibilities and without experiencing excessive physical strain or other distress.
- No contraindication to MRI
  No contraindication to TMS
- Must be free from major medical or neurological conditions unrelated to the pain condition.
- Not Scheduled for total knee replacement surgery (TKR) within 1 month of study visits or other considerable treatment change
- Not on unstable pain medication over the past 4 weeks, or on centrally active medication other than stable antidepressants or on opioidergic analgesic treatment.
- Not on medication, alcohol or recreational drugs though to increase risk of seizure and /or syncope.
- Frequent headaches

Adherence, withdrawal and pausing of study trial

Adherence in this study will be measured by participants' attendance at study visits, completion of all study measures and procedures, and TMS treatments. We will also record the tolerability of 80% RMT and any necessary adjustments of %RMT and NIBS stimulation due to discomfort. Safety instructions also include not being intoxicated by alcohol or non-prescription drugs at any study visit, and any history of heavy binges of alcohol or intermittent hypnotic or benzodiazepine use in the 24 hours before a treatment session because of these behaviours would increase the risk of seizures or syncope with TMS.

Participants who fail to comply with safety instructions can be withdrawn from the study at any time.

Ethics and Trial registration

For peer review only - http://bmjopen.bmj.com/site/about/guidelines.xhtml

All participants will provide written informed consent for taking part in the study. Ethical approval has been obtained by the South West - Cornwall & Plymouth Research Ethics Committee (REC reference:21/SW/0079). For the use of clinical facilities, approval has been obtained from Nottingham University Hospital NHS Trust (RAS 298509 Confirmation of Capacity and Capability).

The trial has been registered (Clinical Trial Reference: ISRCTN15404076, https://www.isrctn.com/ISRCTN15404076).

Known Side Effects and Discomfort

The staff operating the TMS device are trained and adhere to all safety instructions.
As it directly influences activity of nerve cells, theoretically there is the risk of a seizure although the risk level is considered low. The cumulative number of TMS pulses is 36,000 which is substantially lower than the accelerated Stanford protocol and lower than the FDA approved protocol (48,000-72,000 for 4-6 weeks).

Staff and participants will be wearing ear protectors because of the risk of tinnitus and hearing impairment form the loudness of the TMS stimulus.

The majority of TMS treatments are very well tolerated, occasionally however a short-lasting headache can occur. Depending on an individual’s facial anatomy, facial nerves can be stimulated at the same time which would result in muscle twitches in the face; these however cease as the stimulation is stopped. Should a participant experience any side effects and find it too uncomfortable, the treatment session can be stopped immediately but where possible, adjustments will be made to ease discomfort.

Safety assessment

Despite the safety report on accelerated iTBS protocols, side-effects and tolerability in a chronic pain population were unknown, hence the trial was monitored with the intention to stop should larger than expected number of side effects be reported or low compliance be achieved. Within the first 10 participants we did not record any serious side effects nor withdrawal due to protocol non-compliance.

Feedback from Public and patient involvement (PPI)- a lay interest group for clinical research

Patients and healthy lay persons interested in clinical research were invited to give feedback on the

For peer review only - http://bmjopen.bmj.com/site/about/guidelines.xhtml

study concept and separately the specific protocol. Participants of the first PPI were positive about the possibility of a non-invasive treatment that may allow to replace medication. For the specific study protocol, PPI participants indicated that attending the TMS part with its 6 hours long visits for 4 days during a week would be a considerable burden for them, especially for those who suffer from pain-related partial disabilities. Other feedback criticized that this is only open for patients who are mobile, not actively working or on long term sick leave, and that a study of such dimension would need to include inconvenience allowances for participants. We hence included failure to recruit and protocol compliance in the feasibility and acceptability assessment.

Sample size

The pilot aims to recruit n= 45 (30 active, 15 sham iTBS) participants. In this pilot study, we estimate the variance of the outcome measures, and adjust for the upper confidence level (24).

Handling and storage of data

Hardcopies of consent, notes, and data will be stored securely in lockable closets. Digital data will be handled and stored safely on university-owned password-protected servers.

Data analysis

Brain imaging changes will be assessed using current version of standardised and well documented image analysis such as the NIHR Nottingham BRC imaging pipeline (https://github.com/SPMIC-UoN) that has implemented and combined routines from the most suitable advanced image analysis packages, such as FSL, MRConn and the specific protocol with statistical analysis plan will be documented before data analysis will occur.

Discussion

There is an urgent need for innovative treatment options in chronic pain that target the central pain sensitisation and associated maladaptive brain. Osteoarthritis (OA) is a major cause of disability and knee osteoarthritis pain is one of the most common aetiologies of chronic pain affecting about 10% of older adults 55 years of age (25). Chronic knee OA pain is characterised by increased negative affect, enhanced pain sensitivity (25), and impaired central pain modulation (26).

Non-invasive brain stimulation (NIBS) may provide an effective novel approach to reverse brain connectivity changes and associated impaired central pain modulation. While NIBS has proven efficacy in depression (27) results in chronic pain are inconsistent calling for more high-quality research. The

For peer review only - http://bmjopen.bmj.com/site/about/guidelines.xhtml

mixed results to date require systematic piloting of an innovative mechanistic approach before considering well designed clinical trials. The rationale for this protocol was to combine recent promising technical innovations with a focus on the left DLPFC with strongest effective connectivity to the right anterior insula in a target population of moderate chronic knee OA pain with high negative affect scores. Specifically, we combine personalised connectivity-guided target-selection (based on the process by Pszczolkowski et al. (13), intermittent theta burst stimulation, as in our pilot in treatment-resistant depression (28) that is currently undergoing a full RCT evaluation (29), with an accelerated protocol with several daily treatment sessions, adapted from a recently reported intense schedule in treatment-resistant depression (12), with mechanistic neuroimaging and pain assessments. We reasoned that this approach will more effectively 'reset' the altered right anterior insula connectivity changes than previous protocols with further subsequent improvement of central pain modulation and affect. Findings on feasibility, tolerability and exploratory mechanistic effects as well as explorative pain and affect scores will inform on the design, outcome metrics and sample size of future clinical trials.

The main limitations are the pilot design with uncertainty regarding feasibility, tolerability, acceptability and variance of the putative mechanistic intervention. The pilot study aims to assess efficacy to estimate whether such a patient cohort experiences clinically significant pain reduction from this protocol, the extent of potential benefit beyond pain relief, risks, and practical feasibility for patients. This is needed to address feedback from lay interest groups, but also highlighting the potentially significant challenges and barriers for patients with pain-related disabilities to undergo this time-intense treatment protocol at the hospital. Recruitment and completion rates as well as participants' feedback will hence be closely monitored.

For peer review only

Authors' contributions:

MMD performed extensive literature review of TMS pain studies and connectivity analyses, suggested part of the questionnaires, obtained ethical approval and liaised with clinical and regulatory bodies for study set up, negotiated space within suitable facilities, prepared physical setup, purchased study necessities, assisted with training, and organized feedback from lay interest groups.

DPA conceived the study, hypotheses, and designed the study protocol.

DH designed the CPM assessment in this study, adapted MRI scanning protocols, advised on TMS and coil positioning, and takes care of day-to-day study governance.

SH contributes to the trial as research nurse.

BM contributed to the recruitment process design, prepared the database and paper/ electronic questionnaires and assisted with data collection.

STL provided feedback on treatment protocol.

RT contributed to the recruitment process review, safety medical review, and suggested part of the questionnaires.

SP contributed in writing the software for imaging-based personalised connectivity-guided target-selection and manuscript preparation.

For peer review only - http://bmjopen.bmj.com/site/about/guidelines.xhtml

CT provided support with sample size estimations.
DAW provided funds for the trial through his grant from Versus Arthritis UK and provided feedback on the draft manuscript before submission.
MMD & DPA prepared the manuscript. All co-authors edited the draft before submission.

Funding statement: This trial is funded by Versus Arthritis UK and NIHR Nottingham BRC Nottingham. RT received support in part from the UK MRC (CARP MR/T024402/1). None of the funders have active roles in the design of the study, writing of the report, nor are involved in decisions regarding publications.

Competing interests statement: There are no competing interests to declare from any author.

For peer review only

References

1. Malfliet A, Coppieters I, Van Wilgen P, Kregel J, De Pauw R, Dolphens M, et al. Brain changes associated with cognitive and emotional factors in chronic pain: A systematic review. Eur J Pain. 2017;21(5):769-86.
2. Cottam WJ, Iwabuchi SJ, Drabek MM, Reckziegel D, Auer DP. Altered connectivity of the right anterior insula drives the pain connectome changes in chronic knee osteoarthritis. Pain. 2018;159(5):929-38.
3. Cruccu G, Garcia-Larrea L, Hansson P, Keindl M, Lefaucheur JP, Paulus W, et al. EAN guidelines on central neurostimulation therapy in chronic pain conditions. Eur J Neurol. 2016;23(10):1489-99.
4. O'Connell NE, Marston L, Spencer S, DeSouza LH, Wands BM. Non-invasive brain stimulation techniques for chronic pain. Cochrane Db Syst Rev. 2018(3).
5. Saltychev M, Laimi K. Effectiveness of repetitive transcranial magnetic stimulation in patients with fibromyalgia: a meta-analysis. Int J Rehabil Res. 2017;40(1):11-8.
6. Yang SY, Chang MC. Effect of Repetitive Transcranial Magnetic Stimulation on Pain Management: A Systematic Narrative Review. Frontiers in Neurology. 2020;11.
7. Lefaucheur JP, Aleman A, Baeken C, Benninger DH, Brunelin J, Di Lazzaro V, et al. Evidence-based guidelines on the therapeutic use of repetitive transcranial magnetic stimulation (rTMS): An update (2014-2018). Clin Neurophysiol. 2020;131(2):474-528.
8. Galhardoni R, Correia GS, Araujo H, Yeng LT, Fernandes DT, Kaziyama HH, et al. Repetitive Transcranial Magnetic Stimulation in Chronic Pain: A Review of the Literature. Archives of physical medicine and rehabilitation. 2015;96(4):S156-S72.
9. Blumberger DM, Vila-Rodriguez F, Thorpe KE. Effectiveness of theta burst versus high-frequency repetitive transcranial magnetic stimulation in patients with depression (THREE-D): a randomised non-inferiority trial (vol 391, pg 1683, 2018). Lancet. 2018;391(10139):E24-E.
10. Fitzgerald PB. An update on the clinical use of repetitive transcranial magnetic stimulation in the treatment of depression. J Affect Disord. 2020;276:90-103.
11. Duprat R, Desmyter S, De Raedt R, van Heeringen K, Van den Abbeele D, Tandt H, et al. Accelerated intermittent theta burst stimulation treatment in medication-resistant major depression: A fast road to remission? J Affect Disord. 2016;200:6-14.
12. Cole EJ, Stimpson KH, Bentzley BS, Gulser M, Cherian K, Tischler C, et al. Stanford Accelerated Intelligent Neuromodulation Therapy for Treatment-Resistant Depression. Am J Psychiat. 2020;177(8):716-26.
13. Pszczolkowski S, Cottam WJ, Briley PM, Iwabuchi SJ, Kaylor-Hughes C, Shalabi A, et al. Connectivity-Guided Theta Burst Transcranial Magnetic Stimulation Versus Repetitive Transcranial Magnetic Stimulation for Treatment-Resistant Moderate to Severe Depression: Magnetic Resonance Imaging Protocol and SARS-CoV-2-Induced Changes for a Randomized Double-blind Controlled Trial. JMIR Res Protoc. 2022;11(1).

For peer review only - http://bmjopen.bmj.com/site/about/guidelines.xhtml

14. Fox MD, Buckner RL, White MP, Greicius MD, Pascual-Leone A. Efficacy of Transcranial Magnetic Stimulation Targets for Depression Is Related to Intrinsic Functional Connectivity with the Subgenual Cingulate. Biol Psychiat. 2012;72(7):595-603.
15. Kessler S, Grammozis A, Gunther KP, Kirschner S. The Intermittent and Constant Pain Score (ICOAP) - a Questionnaire to Assess Pain in Patients with Gonarthritis. Z Orthop Unfallchir. 2011;149(1):22-6.
16. Akin-Akinyosoye K, James RJE, McWilliams DF, Millar B, das Nair R, Ferguson E, et al. The Central Aspects of Pain in the Knee (CAP-Knee) questionnaire; a mixed-methods study of a self-report instrument for assessing central mechanisms in people with knee pain. Osteoarthr Cartilage. 2021;29(6):802-14.
17. Freynhagen R, Baron R, Gockel U, Tolle TR. painDETECT: a new screening questionnaire to identify neuropathic components in patients with back pain. Curr Med Res Opin. 2006;22(10):1911-20.
18. Sullivan MJL, Bishop SR, Pivik J. The Pain Catastrophizing Scale: Development and validation. Psychol Assessment. 1995;7(4):524-32.
19. Williams N. The GAD-7 questionnaire. Occup Med-Oxford. 2014;64(3):224-.
20. Henry JD, Crawford JR. The short-form version of the Depression Anxiety Stress Scales (DASS-21): construct validity and normative data in a large non-clinical sample. Br J Clin Psychol. 2005;44(Pt 2):227-39.
21. Kenn C, Wood H, Kucyj M, Wattis J, Cunane J. Validation of the Hospital Anxiety and Depression Rating-Scale (Hads) in an Elderly Psychiatric Population. Int J Geriatr Psych. 1987;2(3):189-93.
22. Lowe B, Kroenke K, Herzog W, Grafe K. Measuring depression outcome with a brief self-report instrument: sensitivity to change of the Patient Health Questionnaire (PHQ-9). J Affect Disord. 2004;81(1):61-6.
23. Millar B, McWilliams DF, Abhishek A, Akin-Akinyosoye K, Auer DP, Chapman V, et al. Investigating musculoskeletal health and wellbeing; a cohort study protocol. BMC Musculoskelet Disord. 2020;21(1).
24. Whitehead AL, Julious SA, Cooper CL, Campbell MJ. Estimating the sample size for a pilot randomised trial to minimise the overall trial sample size for the external pilot and main trial for a continuous outcome variable. Stat Methods Med Res. 2016;25(3):1057-73.
25. Peat G, McCarney R, Croft P. Knee pain and osteoarthritis in older adults: a review of community burden and current use of primary health care. Ann Rheum Dis. 2001;60(2):91-7.
26. Petersen KK, McPhee ME, Hoegh MS, Graven-Nielsen T. Assessment of conditioned pain modulation in healthy participants and patients with chronic pain: manifestations and implications for pain progression. Curr Opin Support Palliat Care. 2019;13(2):99-106.
27. Perera T, George MS, Grammer G, Janicak PG, Pascual-Leone A, Wirecki TS. The Clinical TMS Society Consensus Review and Treatment Recommendations for TMS Therapy for Major Depressive Disorder. Brain Stimul. 2016;9(3):336-46.
28. Iwabuchi SJ, Auer DP, Lankappa ST, Palaniyappan L. Baseline effective connectivity predicts response to repetitive transcranial magnetic stimulation in patients with treatment-resistant depression. Eur Neuropsychopharm. 2019;29(5):681-90.
29. Morriss R, Webster L, Abdelghani M, Auer DP, Barber S, Bates P, et al. Connectivity guided theta burst transcranial magnetic stimulation versus repetitive transcranial magnetic stimulation for treatment-resistant moderate to severe depression: study protocol for a randomised double-blind controlled trial (BRIGhTMIND). BMJ Open. 2020;10(7).

For peer review only - http://bmjopen.bmj.com/site/about/guidelines.xhtml

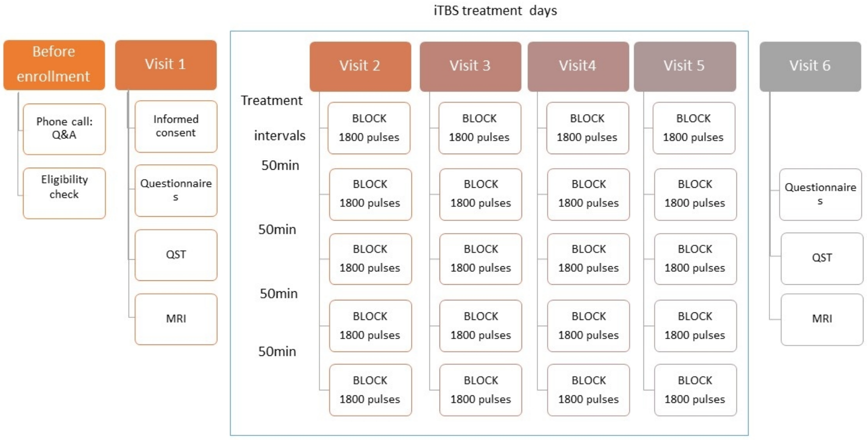

Figure 1: Pilot study overview

338x190mm (96 x 96 DPI)

SPIRIT

STANDARD PROTOCOL ITEMS: RECOMMENDATIONS FOR INTERVENTIONAL TRIALS

SPIRIT 2013 Checklist: Recommended items to address in a clinical trial protocol and related documents*

Section/item	Item No	Description
**Administrative information**		
Title	1	Descriptive title identifying the study design, population, interventions, and, if applicable, trial acronym
Trial registration	2a	Trial identifier and registry name. If not yet registered, name of intended registry
	2b	All items from the World Health Organization Trial Registration Data Set
Protocol version	3	Date and version identifier
Funding	4	Sources and types of financial, material, and other support
Roles and responsibilities	5a	Names, affiliations, and roles of protocol contributors
	5b	Name and contact information for the trial sponsor
	5c	Role of study sponsor and funders, if any, in study design; collection, management, analysis, and interpretation of data; writing of the report; and the decision to submit the report for publication, including whether they will have ultimate authority over any of these activities
	5d	Composition, roles, and responsibilities of the coordinating centre, steering committee, endpoint adjudication committee, data management team, and other individuals or groups overseeing the trial, if applicable (see Item 21a for data monitoring committee)
**Introduction**		
Background and rationale	6a	Description of research question and justification for undertaking the trial, including summary of relevant studies (published and unpublished) examining benefits and harms for each intervention
	6b	Explanation for choice of comparators
Objectives	7	Specific objectives or hypotheses
Trial design	8	Description of trial design including type of trial (eg, parallel group, crossover, factorial, single group), allocation ratio, and framework (eg, superiority, equivalence, noninferiority, exploratory)

For peer review only

Methods: Participants, interventions, and outcomes

Study setting	9	Description of study settings (eg, community clinic, academic hospital) and list of countries where data will be collected. Reference to where list of study sites can be obtained
Eligibility criteria	10	Inclusion and exclusion criteria for participants. If applicable, eligibility criteria for study centres and individuals who will perform the interventions (eg, surgeons, psychotherapists)
Interventions	11a	Interventions for each group with sufficient detail to allow replication, including how and when they will be administered
	11b	Criteria for discontinuing or modifying allocated interventions for a given trial participant (eg, drug dose change in response to harms, participant request, or improving/worsening disease)
	11c	Strategies to improve adherence to intervention protocols, and any procedures for monitoring adherence (eg, drug tablet return, laboratory tests)
	11d	Relevant concomitant care and interventions that are permitted or prohibited during the trial
Outcomes	12	Primary, secondary, and other outcomes, including the specific measurement variable (eg, systolic blood pressure), analysis metric (eg, change from baseline, final value, time to event), method of aggregation (eg, median, proportion), and time point for each outcome. Explanation of the clinical relevance of chosen efficacy and harm outcomes is strongly recommended
Participant timeline	13	Time schedule of enrolment, interventions (including any run-ins and washouts), assessments, and visits for participants. A schematic diagram is highly recommended (see Figure)
Sample size	14	Estimated number of participants needed to achieve study objectives and how it was determined, including clinical and statistical assumptions supporting any sample size calculations
Recruitment	15	Strategies for achieving adequate participant enrolment to reach target sample size

Methods: Assignment of interventions (for controlled trials)

Allocation:

Sequence generation	16a	Method of generating the allocation sequence (eg, computer-generated random numbers), and list of any factors for stratification. To reduce predictability of a random sequence, details of any planned restriction (eg, blocking) should be provided in a separate document that is unavailable to those who enrol participants or assign interventions

Allocation concealment mechanism	16b	Mechanism of implementing the allocation sequence (eg, central telephone; sequentially numbered, opaque, sealed envelopes), describing any steps to conceal the sequence until interventions are assigned
Implementation	16c	Who will generate the allocation sequence, who will enrol participants, and who will assign participants to interventions
Blinding (masking)	17a	Who will be blinded after assignment to interventions (eg, trial participants, care providers, outcome assessors, data analysts), and how
	17b	If blinded, circumstances under which unblinding is permissible, and procedure for revealing a participant's allocated intervention during the trial
**Methods: Data collection, management, and analysis**		
Data collection methods	18a	Plans for assessment and collection of outcome, baseline, and other trial data, including any related processes to promote data quality (eg, duplicate measurements, training of assessors) and a description of study instruments (eg, questionnaires, laboratory tests) along with their reliability and validity, if known. Reference to where data collection forms can be found, if not in the protocol
	18b	Plans to promote participant retention and complete follow-up, including list of any outcome data to be collected for participants who discontinue or deviate from intervention protocols
Data management	19	Plans for data entry, coding, security, and storage, including any related processes to promote data quality (eg, double data entry; range checks for data values). Reference to where details of data management procedures can be found, if not in the protocol
Statistical methods	20a	Statistical methods for analysing primary and secondary outcomes. Reference to where other details of the statistical analysis plan can be found, if not in the protocol
	20b	Methods for any additional analyses (eg, subgroup and adjusted analyses)
	20c	Definition of analysis population relating to protocol non-adherence (eg, as randomised analysis), and any statistical methods to handle missing data (eg, multiple imputation)
**Methods: Monitoring**		
Data monitoring	21a	Composition of data monitoring committee (DMC); summary of its role and reporting structure; statement of whether it is independent from the sponsor and competing interests; and reference to where further details about its charter can be found, if not in the protocol. Alternatively, an explanation of why a DMC is not needed

	21b	Description of any interim analyses and stopping guidelines, including who will have access to these interim results and make the final decision to terminate the trial
Harms	22	Plans for collecting, assessing, reporting, and managing solicited and spontaneously reported adverse events and other unintended effects of trial interventions or trial conduct
Auditing	23	Frequency and procedures for auditing trial conduct, if any, and whether the process will be independent from investigators and the sponsor
**Ethics and dissemination**		
Research ethics approval	24	Plans for seeking research ethics committee/institutional review board (REC/IRB) approval
Protocol amendments	25	Plans for communicating important protocol modifications (eg, changes to eligibility criteria, outcomes, analyses) to relevant parties (eg, investigators, REC/IRBs, trial participants, trial registries, journals, regulators)
Consent or assent	26a	Who will obtain informed consent or assent from potential trial participants or authorised surrogates, and how (see Item 32)
	26b	Additional consent provisions for collection and use of participant data and biological specimens in ancillary studies, if applicable
Confidentiality	27	How personal information about potential and enrolled participants will be collected, shared, and maintained in order to protect confidentiality before, during, and after the trial
Declaration of interests	28	Financial and other competing interests for principal investigators for the overall trial and each study site
Access to data	29	Statement of who will have access to the final trial dataset, and disclosure of contractual agreements that limit such access for investigators
Ancillary and post-trial care	30	Provisions, if any, for ancillary and post-trial care, and for compensation to those who suffer harm from trial participation
Dissemination policy	31a	Plans for investigators and sponsor to communicate trial results to participants, healthcare professionals, the public, and other relevant groups (eg, via publication, reporting in results databases, or other data sharing arrangements), including any publication restrictions
	31b	Authorship eligibility guidelines and any intended use of professional writers
	31c	Plans, if any, for granting public access to the full protocol, participant-level dataset, and statistical code

For peer review only

Appendices

Informed consent materials	32	Model consent form and other related documentation given to participants and authorised surrogates
Biological specimens	33	Plans for collection, laboratory evaluation, and storage of biological specimens for genetic or molecular analysis in the current trial and for future use in ancillary studies, if applicable

*It is strongly recommended that this checklist be read in conjunction with the SPIRIT 2013 Explanation & Elaboration for important clarification on the items. Amendments to the protocol should be tracked and dated. The SPIRIT checklist is copyrighted by the SPIRIT Group under the Creative Commons "Attribution-NonCommercial-NoDerivs 3.0 Unported" license.

For peer review only

BMJ Open

Brain connectivity-guided, optimised theta burst transcranial magnetic stimulation to improve central pain modulation in knee osteoarthritis pain (BoostCPM): protocol of a pilot randomised clinical trial in a secondary care setting.

Journal:	*BMJ Open*
Manuscript ID	bmjopen-2023-073378.R1
Article Type:	Protocol
Date Submitted by the Author:	13-Jul-2023
Complete List of Authors:	Drabek, Marianne; University of Nottingham,<br>Hodkinson, Duncan; Nottingham NIHR Biomedical Research Centre; University of Nottingham<br>Horvath, Suzanne; Nottingham NIHR Biomedical Research Centre<br>Millar, Bonnie; University of Nottingham Faculty of Medicine and Health Sciences, Academic Rheumatology; NIHR Nottigham Biomedical Research Centre, Academic Rheumatology<br>Pszczolkowski Parraguez, Stefan; University of Nottingham<br>Tench, Christopher; University of Nottingham, Division of Clinical Neuroscience<br>Tanasescu, Radu; University of Nottingham,<br>Lankappa, Sudheer; Nottinghamshire Healthcare NHS Foundation Trust, Adult Mental Health<br>Morriss, Richard; University of Nottingham, Psychiatry<br>Walsh, David; University of Nottingham, Academic Rheumatology<br>Auer, Dorothee; Nottingham NIHR Biomedical Research Centre; University of Nottingham
**Primary Subject Heading**:	Mental health
Secondary Subject Heading:	Rheumatology
Keywords:	Chronic Pain, PAIN MANAGEMENT, PSYCHIATRY

SCHOLARONE™
Manuscripts

Brain connectivity-guided, optimised theta burst transcranial magnetic stimulation to improve central pain modulation in knee osteoarthritis pain (BoostCPM): protocol of a pilot randomised clinical trial in a secondary care setting.

Authors

Marianne M. Drabek[1,2,3], Duncan Hodkinson[1,2,3], Suzanne Horvath[1], Bonnie Millar[1,3], Stefan Pszczolkowski[1,2], Christopher Tench[1,2, 5], Radu Tanasescu[1,4,5], Sudheer T Lankappa[6], Richard Morriss[1], David A. Walsh[3], Dorothee P. Auer[1,2,3]

Affiliations

[1] Nottingham NIHR Biomedical Research Centre

[2] Sir Peter Mansfield Imaging Centre, School of Medicine, University of Nottingham, Nottingham

[3] Pain Centre Versus Arthritis, University of Nottingham, Nottingham

[4] Department of Neurology, Queen's Medical Centre, Nottingham University Hospital Trust

[5]Clinical Neurology, School of Medicine, University of Nottingham

[6]Psychiatry, Nottinghamshire Healthcare Foundation Trust

Corresponding author

Dorothee P. Auer, Radiological Sciences, Room W/B 1441, Queen's Medical Centre, Nottingham, NG7 2UH, UK. Email: dorothee.auer@nottingham.ac.uk

Word count: 4059

Key words: Transcranial Magnetic Stimulation, chronic pain, refractory pain, osteoarthritis, brain connectivity, dorsolateral prefrontal cortex

For peer review only - http://bmjopen.bmj.com/site/about/guidelines.xhtml

For peer review only

Abstract

Introduction

Chronic pain is a common health problem that is not efficiently managed by standard analgesic treatments. There is evidence that treatment resistance may result from maladaptive brain changes in areas that are fundamental to the perception of pain. Knee osteoarthritis is one of the most prevalent causes of chronic pain and commonly associated with negative affect. Chronic knee osteoarthritis pain is also associated with altered right anterior insula functional connectivity. We posit that reversal of these brain circuit alterations may be critical to alleviate chronic pain and associated negative affect, and that this can be achieved through non-invasive neuromodulation techniques. Despite growing interest in non-invasive neuromodulation for pain relief and proven efficacy in depression, results in chronic pain are mixed with limited high-quality evidence for clinical and mechanistic efficacy. Limitations include patient heterogeneity, imprecision of target selection, uncertain blinding and protocols that may deliver pulses at subclinical efficacy.

Methods and Analysis

We hence developed an optimised treatment protocol of connectivity-guided intermittent theta-burst stimulation (iTBS) targeting the left dorso-lateral prefrontal cortex with accelerated delivery on four consecutive days (allowing four days within the same week as protocol variation) with 5 daily treatment sessions that will be piloted in a sham-controlled design in 45 participants with chronic knee pain. This pilot study protocol will assess feasibility, tolerability and explore mechanistic efficacy through serial functional/structural MRI and quantitative sensory testing.

Ethics and Dissemination

This pilot trial has been approved by the Ethics Committee Cornwall & Plymouth.
Results of the pilot trial will be submitted to peer-reviewed journals, presented at research conferences and may be shared with participants and PPI/E advisors.

Trial registration

The trial has been registered (Clinical Trial Reference: ISRCTN15404076)

For peer review only

For peer review only - http://bmjopen.bmj.com/site/about/guidelines.xhtml

Strengths and limitations of this study

- The protocol combines connectivity-guided neuronavigation for improved precision to target the prefronto-insular circuit with accelerated delivery of five daily iTBS sessions/blocks over four days in one week for enhanced efficacy and efficiency in a sham-controlled RCT design.
- Advanced serial MRI of brain network function complements psychophysical and questionnaire outcome markers before and after TMS protocol to explore mechanistic efficacy.
- Recruitment is predominantly from a research-readycommunity cohort which together with the single centre design may limit generalisability.
- The intensive protocol may only be accommodated and tolerated by a subgroup of pain patients.
- The total number of 36,000 pulses and the permitted reduction of RMT below 80% may limit efficacy of the intervention.

Introduction

Chronic pain is a major burden for society, economy, health systems and individuals. There is a lack of effective analgesic treatments in chronic pain with an urgent need for novel treatments. Maladaptive brain mechanisms are increasingly recognised as key contributors to the development and persistence of pain (1). A feature is the altered communication between brain areas that are involved in the processing of pain signals and the modulation of the associated pain perception, demonstrated by dysfunctional connectivity of the right anterior insula (2).

These maladaptive functional brain network changes are thought (i) to drive the experience of chronic pain and central pain sensitisation, (ii) to be potentially reversible by non-invasive neuromodulation, and thus support the notion that interventions that normalise the brain's (and in particular fronto-limbic) connectivity will also reverse central pain augmentation or associated affective experience.

Repetitive transcranial Magnetic Stimulation (rTMS) is a promising tool to non-invasively and focally stimulate the brain through induction of small electrical currents. Choice of stimulation protocols are thought to determine excitatory or inhibitory local and downstream effects with acute and longer-term neuromodulatory effects on targeted brain circuits. The exact mechanism of acute neuromodulatory and neuroplastic effects and the substantial inter-and intraindividual variability are poorly understood.(3).Clinical efficacy of rTMS is well established for the treatment of major depression, approved by the Federal Drug Administration (FDA) in the US and the National Institute of Clinical Excellence (NICE) in the UK. In chronic pain, to date evidence for effective neuromodulation is limited to invasive protocols and selected indications (4). There is, however,

For peer review only - http://bmjopen.bmj.com/site/about/guidelines.xhtml

considerable recent interest in non-invasive brain stimulation (NIBS) trials in chronic pain yet results to date are mixed.

Meta-analyses and systematic reviews have described NIBS as having 'poor or inconclusive' analgesic effects across sites and pain disorders (4-6), "minimally clinically relevant" (6), to "promising"(7) and "probable efficacy" for either motor cortex (M1) or dorsolateral prefrontal cortex (DLPFC) stimulation (8). There are major quality concerns with most studies considered poor (3) due to underpowered sample sizes, lack of blinding, inadequate randomization, or missing sham for comparisons. Moreover, there are uncertainties regarding the most effective brain stimulation site, type of stimulation, target population. Nevertheless, pain relief was frequently found to be >30% (9) which encourages to seek further improvements and address limitations in order to fully assess and exploit the potential that this technology may hold for pain treatment.

To address these shortcomings we chose to target the left DLPFC as a particularly promising yet relatively understudied target site for rTMS in chronic pain due to its strong modulatory influence on the right anterior insula (rAI), a key hub in pain processing with known altered connectivity in chronic pain(2). Stimulating the left DLPFC is expected to modulate the prefronto-insular pathway as a putative central pain modulatory effect. This pathway is also a key target in the clinically effective rTMS treatment of major depression suggesting a second putative beneficial effect through reduction of negative affect and related pain augmentation.

Reliability of the individual target selection is improved by neuronavigation typically based on standard anatomical magnetic resonance imaging (MRI). Further substantive enhancement of target precision can be achieved through the innovative approach of directional connectivity strength analysis (using resting state functional MRI) between the DLPFC and rAI using task-free functional MRI. TMS targeted to those DLPFC voxels that exert the strongest influence on the rAI is thought to induce a stronger on the DLPFC-rAI circuitry and rAI. Hence, we plan to use resting state functional MRI based effective connectivity to determine the coordinates within the anatomically defined left DLPFC that show the strongest effective connectivity to the right anterior insula in our previous work (10, 11).

In addition, recently accelerated, optimised TMS protocols were reported to be safe and similarly effective to standard rTMS using intermittent theta burst stimulation (iTBS)(12-15). There are several advantages expected from iTBS protocols that are faster than standard rTMS and may have stronger neuroplasticity effects. Also, the multiple treatment blocks per day with 50 minutes intervals are expected to further enhance effectiveness and allow to deliver a higher cumulative weekly dose with the benefit of shorter overall treatment course.

For peer review only - http://bmjopen.bmj.com/site/about/guidelines.xhtml

Motivated by the promising results of the optimised iTBS protocol in major depression, we translated and adapted the approach to chronic musculo-skeletal pain, which, if effective, would greatly improve clinical interest in this technique both for cost and practical reasons. As study population we chose knee osteoarthritis (OA) as one of the most common causes of chronic musculo-skeletal pain and to avoid potential heterogeneity from different pain aetiology. Based on the known comorbidity, we anticipate that the study population of moderate chronic knee OA pain will report higher negative affect than the general population which may further increase the likelihood of neuromodulatory efficacy at the chosen target site.

This parallel, sham-controlled pilot study will investigate a four day (over one week) intense, imaging-guided intermittent theta burst stimulation protocol that is individually targeted within the left DLPFC to maximise the connectivity with the rAI. Study participants with moderately severe, chronic knee pain will undergo serial MRI, behavioural and quantitative sensory testing before and after the iTBS/sham intervention and online recording of patient-reported outcomes until week 18 to guide the protocol and sample size for future RCT interventions.

Objectives

(i) To test the feasibility and tolerability of an optimised accelerated four day (over one week) iTBS protocol with 5 daily sessions (total pulses 36,000 aimed at 80% RMT) using connectivity-guided targeting of the left DLPFC in chronic knee OA patients with moderately severe pain and self-reported negative affect.

(ii) To assess the variance of mechanistic outcome markers comparing the intense iTBS protocol versus sham.

(iii) To explore clinical efficacy metrics, and the feasibility of online recorded patient-reported outcome measures in the iTBS and sham arm.

(iv) To explore the effects of iTBS on negative affect, stress and arousal between iTBS and sham.

(v) To assess feasibility of blinding using sham TMS.

Methods and Analysis

Trial design

Single centre, sham controlled randomised mechanistic pilot study at the University of Nottingham with a parallel group study design, whereby participants either receive active iTBS or sham iTBS with random allocation of 2:1. The uneven allocation was chosen to achieve blinding but preferencing power for statistical analyses in the active condition.

For peer review only - http://bmjopen.bmj.com/site/about/guidelines.xhtml

Hypotheses

Primary mechanistic hypotheses

1. Accelerated, personalised iTBS of the DLPFC modulates *functional activity and connectivity* of brain circuits involved in central pain control in chronic musculoskeletal pain compared with sham controls. The direction of change is expected to reduce hallmark features of the pain connectome, such as reduction of the abnormally increased rAI-posterior default mode network anticorrelation.
2. Accelerated, personalised iTBS of the DLPFC increases endogenous antinociception in chronic MSK pain compared to sham controls.

Secondary Hypotheses

3. Accelerated, personalised iTBS of the DLPFC reduces pain catastrophizing, anxiety and improves mood in chronic musculoskeletal pain compared to sham controls.
4. Accelerated, personalised iTBS of the DLPFC reduces sensitivity to experimental pain at the end of the intervention compared to sham controls.
5. Accelerated, personalised iTBS of the DLPFC reduces physiological arousal and reported stress levels compared to sham controls.

Study Procedures

Participants will first be asses

sed over phone for meeting safety and eligibility criteria before giving informed written consent.

Participants will be asked to attend for baseline assessments (visit 1) (ca. 2 hours including MRI, and psycho-physical assessment including brief experimental pain exposure) (see Figure 1). Then participants will undergo an intervention week with four visits (Visit 2-5) lasting about half a day each. Each visit will consist of five blocks of iTBS or sham with inter-block interval of ~50 minutes during which participants will be offered refreshments and food in a comfortable waiting area before getting prepared for the next session. The 6th and final study visit is a repeat of the baseline visit, and will be scheduled within 18 -72 hours of the last treatment session. This will be followed by online patient self-recording of pain and emotional outcomes up to 16 weeks.

Recruitment and inclusion criteria

For peer review only - http://bmjopen.bmj.com/site/about/guidelines.xhtml

Participants will be recruited primarily from previous participants who consented to be contacted (such as the community-based Investigating Musculoskeletal Health and Wellbeing cohort) (23) and additional recruitment pathways via posters, general practitioner surgeries and other NHS services (Joint replacement waiting list) or co-recruited from ongoing studies that allow co-recruitment as required. Individuals who consented to be contacted about new studies, will be contacted by the study team who originally consented the participant; only if the participant then expresses interest in the new study and agrees for their contact details to be passed on to the new study team, the latter will contact them.

Participants may be initially invited to take part in the study by sending a short format participant information sheet (PIS) form that summarizes the main points of the study for consideration of interest; participants who contact for further information will be sent the final PIS. Alternatively, they may find the short format PIS as a poster and contact us. Posters may be put around the Nottingham University Hospital Trust, the Medical School and other University of Nottingham sites, public sites such as public libraries and leisure/community centres. Posters may also be placed on local press, including but not limited to The Nottingham Post and Metro. The same advert may be featured on UK websites related to chronic pain, including but not limited to the Pain Centre Versus Arthritis webpages within http://www.nottingham.ac.uk/paincentre.

Participant enrolment and retention will be continually monitored, and numbers fed back to the study team meetings. Feedback from participants will be used to maintain recruitment and retention rates.

Inclusion criteria

- 18-75 years of age and able to consent
- Chronic knee pain (minimum 6 months duration) with VAS score >=4/10
-
- Able to accommodate the study visits without disruption to their jobs or other responsibilities and without experiencing excessive physical strain or other distress.

Exclusion criteria

Patients are only admitted to the study if they also meet the following criteria:

- No contraindication to MRI and TMS

- No major medical or neurological conditions unrelated to the pain condition.

For peer review only - http://bmjopen.bmj.com/site/about/guidelines.xhtml

- Not scheduled for total knee replacement surgery (TKR) within 1 month of study visits or other considerable treatment change
- No change in pain medication in the past four weeks,
- Not on centrally active medication other than stable antidepressants or on opioidergic analgesic treatment.
- Not on medication, alcohol or recreational drugs, thought to increase risk of seizure and /or syncope.
- Not experiencing frequent headaches
-

Participants will be reimbursed for travel expenses and provided with free drinks and food during study sessions but will not be compensated for their time.

Transcranial magnetic stimulation

Transcranial magnetic stimulation (TMS) will be delivered using the Magstim Horizon Performance system with StimGuide Navigated TMS package (The Magstim Co. Ltd). Single-pulse TMS will be applied to the left motor cortex using a handheld figure-of-eight coil (diameter 7cm) to determine the resting motor threshold (RMT). RMT is defined as the minimum stimulator output intensity needed to achieve a minimum motor evoked potential (MEP)-amplitude of 50μV in the completely relaxed abductor pollicis brevis (APB) muscle in at least 5 out of 10 trials. For all treatments, the intermittent theta burst stimulation (iTBS) protocol will be delivered using a fixed 7cm diameter coil (E-z Cool Coil and support arm) consisting of 3 pulses (aimed at 80% RMT, but may be lowered to avoid participant discomfort) of 50Hz repeated at 200ms (5Hz) intervals with a total 1800 pulses per block, 9000 pulses per visit/day and 36,000 pulses in total/ treatment week.

The staff operating the TMS device (SZ, senior research nurse with 3 years' experience in rTMS treatment/trials; DH senior research fellow with 2 years' experience in research TMS) are trained on the MAGSTIM system and adhere to all safety instructions.

MRI acquisition

Imaging data will be acquired on a GE Premier 3T magnetic resonance scanner using a 48-channel head coil (GE Healthcare, USA). Structural scans include high resolution T1-weighted (T1w) and T2-weighted (T2w) images for the purpose of image registration. The T1w images will be acquired using 3D MPRAGE sequence (voxel size=1mm isotropic, field of view = 256, matrix = 256, 256 sagittal slices, TI = 800 ms, FA = 8°) and T2w will be images acquired using 3D Cube T2 FLAIR sequence (voxel size=1mm isotropic, FOV = 256, matrix = 256, 256 sagittal slices, TR = 6300ms, TE =121ms, TI =1787ms). The resting-state fMRI data will be acquired using whole-brain 2D GE-EPI sequence

For peer review only - http://bmjopen.bmj.com/site/about/guidelines.xhtml

(TR=1400ms, TE=35ms, flip angle=68°, in-plane FOV = 212×212mm, 57 slices, slice thickness=2mm, voxel size=2mm isotropic, hyperband factor=3, ARC factor=2, 15min total scan time). To measure the b_0 field for correction of EPI distortions, we will also acquire two SE-EPI images with reversed phase encoding directions. The SE-EPI images have the same geometry, echo spacing, and phase encoding direction parameters as the GE fMRI scans.

Image processing and TMS neuronavigation

Image pre-processing follows the same procedure to that described in Pszczolkowski et al. (10). In brief, the structural T1-weighted images are brain extracted, bias corrected and segmented into white matter (WM), grey matter (GM) and cerebrospinal fluid (CSF) probability maps which are binarised at a probability of 0.98 and then eroded using a spherical kernel with a radius of 2 voxels to obtain the final tissue labels. The blood oxygenation level dependent resting state images (BOLD rsfMRI) undergo EPI distortion correction, motion correction, slice timing correction, smoothing, denoising and high-pass filtering. Finally, the WM and GM tissue labels are transformed into rsfMRI BOLD space and used to regress out the WM and GM time series from the processed rsfMRI BOLD data.

For identification of the iTBS treatment target, a methodology similar to Pszczolkowski et al. (10) will be followed. However in the current pilot trial the DLPFC searchlight described in Fox et al. (16) will be used as primary volume of interest and that the background removal procedure is based on a threshold computed automatically rather than by visual inspection. This threshold is computed as the 98th percentile of the T1-weighted intensities within a mask of background (air) voxels around the outside of the skull in MNI space which is previously transformed into T1-weighted space.

Quantitative sensory testing (QST)

Central pain inhibition will be tested pre- and post-TBS treatment using the psychophysical paradigm of conditioned pain modulation (CPM). The test stimulus will be pressure pain threshold (PPT) and the conditioning stimulus ischemic pain caused by a blood pressure cuff placed at the calf of the unaffected leg. The test stimulus (PPTs) will be administered at three body sites (on the affected side), including trapezius muscle, metacarpophalageal joint of the thumb, and the patella. If no preference is shown with respect to the affected side, then the patients non-dominant body side will be examined. All QST assessment are completed by DH with > 10 years' experience. If no

For peer review only - http://bmjopen.bmj.com/site/about/guidelines.xhtml

preference is shown with respect to the affected side, then the patients non-dominant body side will be examined. All QST assessment are completed by DH with > 10 years' experience.

Conditioned pain modulation is calculated as follows:

CPM (%) = (PPTpost-conditioning – PPTpre-conditioning)/ PPTpre-conditioning X 100.

Pre-conditioning PPTs will be used to calculate SEM and >±2XSEM to determine CPM effects in response TBS treatment.

Sham, randomisation and blinding

The sham intervention will be given through the same coil as the iTBS treatment and both active and sham will be administered over the left DLPFC using neuronavigation co-ordinates (s. above). For the sham group pulses will be delivered at 30% stimulator output by orienting the coil perpendicular to the scalp surface with direct contact. This will ensure that an appropriate audible noise is created to reinforce the integrity of the treatment blinding.The study statistician will generate allocation by randomising blocks of participants, ensuring age and sex matching between arms. Block randomisation (block size 6) will be used determined using online tools (http://www.jerrydallal.com/random/randomize.htm). Initially 30 subjects will be randomised with 3 subjects per group in each block. Given the recruitment rates and feasibility of recruiting all subjects within the time frame, the next 30 subjects will be randomised similarly. However, if recruitment suggests a shortfall of subjects within the time frame the block allocation will be adjusted such that a higher rate of subjects are recruited to the treatment group such as to ensure sufficient numbers in that group. This is expected to be at most a small adjustment such that the block of 6 will still be sufficient to effectively mask assignment, for example 4 into the treatment group and 2 into the sham group. The study co-ordinator (BM) will enrol and assign participants.

Study participants will be blinded to the intervention allocation and participants will be asked whether they can guess the type of intervention as part of an TMS exist interview/questionnaire. The study team involved in delivering the intervention cannot be blinded, but the data analysis team will be blinded to which intervention was delivered. Participants will be unblinded if they were to experience adverse events. *Assessments*

Data will be collected for the following

- MRI at visit 1 and at visit 6 (pre- and post-treatment)
- Quantitative sensory testing (QST) at visit 1 and at visit 6 (pre- and post-treatment)

RedCap (https://www.project-redcap.org/) will be used to administer online questionnaires:

Pain characteristics

- a numerical pain VAS (0-10) as online daily pain report

For peer review only - http://bmjopen.bmj.com/site/about/guidelines.xhtml

- weekly questionnaires:

  Intermittent and Constant Pain Score (ICOAP)(17), The Central Aspects of Pain in the Knee (CAP-Knee) questionnaire (18)
- at visit 1 &6:

  painDETECT (19), Pain Catastrophizing Scale (PCS) (20), ICOAP (17), General Anxiety Depression scale (GAD-7) (21), Depression Anxiety Stress Scales (DASS-21)(22), Hospital Anxiety and Depression Rating-Scale (HADS)(23), Patient Health Questionnaire (PHQ-9)(24), CAP-Knee(18), Montreal Cognitive Assessment (MoCA) Test(25).

Outcome metrics and measures

Outcome metrics are changes between end of the intervention (Visit 6) and baseline (Visit 1) of optimized neuroimaging markers of the DPMS that will be established from independent dataset and pre-defined in the analysis plan and secondly markers of central pain modulation, derived from QST.

Outcome measures

Primary:

1. Variance of changes in pre-selected neuroimaging functional markers of the descending pain modulatory system at final vs baseline visit.
2. Variance of change in conditioned pain modulation (pain intensity change with / without conditioned pain stimulus) at final vs baseline visit.
3. Number of participants recruited and retained on the trial.
4. Number of iTBS sessions completed at 80% RMT.

Secondary:

5. Variance of change in reported pain intensity, burden and catastrophizing at final versus baseline study visit (ICOAP, PCS)
6. Change in negative affect (HADS) at final versus baseline study visit.
7. Variance in physiological and psychophysical changes (acute experimental pain) at final vs baseline study visit.

For peer review only - http://bmjopen.bmj.com/site/about/guidelines.xhtml

8. Variance of PROMS (daily online recording pain, wellbeing, sleep) over 16 weeks from treatment versus enrolment-baseline.

Adherence, tolerability, withdrawal and pausing of study trial

Adherence in this study will be measured by participants' attendance at study visits, completion of all study measures and procedures, and TMS treatments. We will also record the tolerability of 80% RMT and any necessary adjustments of %RMT and NIBS stimulation due to discomfort. Safety instructions also include not being intoxicated by alcohol or non-prescription drugs at any study visit, and any history of heavy binges of alcohol or intermittent hypnotic or benzodiazepine use in the 24 hours before a treatment session because of these behaviours would increase the risk of seizures or syncope with TMS.

Participants who fail to comply with safety instructions can be withdrawn from the study at any time. Study withdrawal could be requested anytime by the participants.Tolerability of the accelerated protocol will be individually monitored in the first 10 participants with planned meeting of the study team with the independent medical safety advisor (RT) for go/no-go decision making should the study retention exceed expected levels for rTMS studies or based on concerning individual feedback. Within the first 10 participants we did not observe withdrawal due to protocol non-compliance.

Known Side Effects and Discomfort

The staff operating the TMS device (SZ 3 years, DH 2 years) are trained and adhere to all safety instructions.

As it directly influences activity of nerve cells, theoretically there is the risk of a seizure although the risk level is considered low. The cumulative number of TMS pulses is 36,000 which is substantially lower than the accelerated Stanford protocol and lower than the FDA approved protocol (48,000-72,000 for 4-6 weeks).

Staff and participants will be wearing ear protectors because of the risk of tinnitus and hearing impairment from the loudness of the TMS stimulus.

The majority of TMS treatments are very well tolerated, occasionally however a short-lasting headache can occur. Depending on an individual's facial anatomy, facial nerves can be stimulated at the same time, which would result in muscle twitches in the face; these however cease as the stimulation is stopped.

For peer review only - http://bmjopen.bmj.com/site/about/guidelines.xhtml

Should a participant experience any side effects and find it too uncomfortable, the treatment session can be stopped immediately but where possible, adjustments will be made to ease discomfort. To reduce participants' discomfort on the first day of the session, the power was individually ramped to 80% RMT or lower depending on subjective tolerability.

A standard operating procedure has been developed that defines the discontinuation of the trial intervention, referral to treatment as required and risk management of the most common serious side effect (seizure) and more common side effects like headache and dizziness.

Safety assessment

Despite the safety report on accelerated iTBS protocols, side-effects and tolerability in a chronic pain population were unknown, hence the trial will be monitored with the intention to stop should a larger than expected number of side effects be reported or low compliance be found. Within the first 10 participants we did not record any serious side effects. Any safety-related termination decision would be taken by the medical safety advisor.

Feedback from Public and patient involvement (PPI)- a lay interest group for clinical research

Patients and healthy lay persons interested in clinical research were invited to give feedback on the study concept and separately the specific protocol. For the specific study protocol, PPI participants indicated that attending the TMS part with its up to 6 hours long visits for 4 days during a week would be a considerable burden for them, especially for those who suffer from pain-related partial disabilities. Other feedback criticised that this is only open for patients who are mobile, not actively working or on long term sick leave, and that a study of such dimension would need to include inconvenience allowances for participants. We hence included failure to recruit and protocol compliance in the feasibility and acceptability assessment.

Sample size

The pilot aims to recruit n= 45 (30 active, 15 sham iTBS) participants. In this pilot study, we estimate the variance of the outcome measures, and adjust for the upper confidence level (26).

Handling and storage of data

Hardcopies of consent, notes, and data will be stored securely in lockable closets. Digital data will be handled and stored safely on university-owned password-protected servers. Data will be entered in a RedCAP database. To maintain data quality one person will enter the data and a second will check

For peer review only - http://bmjopen.bmj.com/site/about/guidelines.xhtml

and validate the data. Data will be audited on a monthly basis and any issues raised at the biweekly study team meetings, one of whose functions is to monitor quality.

Data analysis

Brain imaging changes will be assessed using current version of standardised and well documented image analysis such as the NIHR Nottingham BRC imaging pipeline (https://github.com/SPMIC-UoN) that has implemented and combined routines from the most suitable advanced image analysis packages, such as FSL, MRConn and the specific protocol with statistical analysis plan will be documented before data analysis will occur.

Trial status

The study started in July 2021 until July 2024. The recruitment opened 12/05/2022 and is anticipated to be completed by 31/12/2023. As of March 2023, 28 participants completed the trial.

Given the pilot nature and small size of the trial, no DMC was needed but instead an independent experienced neurologists (RT) acted as safety advisor to optimally manage the seizure risk.

Ethics and Dissemination

All participants will provide written informed consent for taking part in the study. Ethical approval has been obtained by the South West - Cornwall & Plymouth Research Ethics Committee (REC reference:21/SW/0079). For the use of clinical facilities, approval has been obtained from Nottingham University Hospital NHS Trust (RAS 298509 Confirmation of Capacity and Capability).

The trial has been registered (Clinical Trial Reference: ISRCTN15404076, https://www.isrctn.com/ISRCTN15404076).

Results of the feasibility and pilot trial will be submitted for publication in open access, peer-review journals, for presentation at scientific conferences and may be shared with participants and PPI/E advisors.

For peer review only - http://bmjopen.bmj.com/site/about/guidelines.xhtml

Authors' contributions:
MMD & DPA prepared the manuscript and edited the manuscript during rebuttal.
MMD performed extensive literature review of TMS pain studies and functional connectivity analyses, suggested part of the questionnaires, obtained ethical approval and liaised with clinical and regulatory bodies for study set up, negotiated space within suitable facilities, prepared physical setup, purchased study necessities, assisted with training, and organized feedback from lay interest groups.
DPA conceived the study, hypotheses, and designed the study protocol.
DH designed the CPM assessment in this study and performs the assessment, adapted MRI scanning protocols, advised on TMS and coil positioning, takes care of day-to-day study governance, and edited the manuscript during rebuttal.
SH contributes to the trial as research nurse.
BM contributed to the recruitment process design, prepared the database and paper/ electronic questionnaires and assisted with data collection, and edited the manuscript during rebuttal.
STL provided feedback on treatment protocol.
RT contributed to the recruitment process review, safety medical review, and commented on part of the questionnaires.
SP contributed in writing the software for imaging-based personalised connectivity-guided target-selection.
CT provided support with sample size estimations.
DAW provided funds for the trial through his grant from Versus Arthritis UK and provided feedback on the draft manuscript before submission.
All co-authors provided feedback on the draft before submission.

Funding statement: This trial is funded by Versus Arthritis UK and NIHR Nottingham BRC Nottingham. RT received support in part from the UK MRC (CARP MR/T024402/1). None of the funders have active roles in the design of the study, writing of the report, nor are involved in decisions regarding publications.

Competing interests statement: There are no competing interests to declare from any author.

References

1. Malfliet A, Coppieters I, Van Wilgen P, Kregel J, De Pauw R, Dolphens M, et al. Brain changes associated with cognitive and emotional factors in chronic pain: A systematic review. Eur J Pain. 2017;21(5):769-86.
2. Cottam WJ, Iwabuchi SJ, Drabek MM, Reckziegel D, Auer DP. Altered connectivity of the right anterior insula drives the pain connectome changes in chronic knee osteoarthritis. Pain. 2018;159(5):929-38.
3. Jannati A, Oberman LM, Rotenberg A, Pascual-Leone A. Assessing the mechanisms of brain plasticity by transcranial magnetic stimulation. Neuropsychopharmacol. 2023;48(1):191-208.
4. Cruccu G, Garcia-Larrea L, Hansson P, Keindl M, Lefaucheur JP, Paulus W, et al. EAN guidelines on central neurostimulation therapy in chronic pain conditions. Eur J Neurol. 2016;23(10):1489-99.
5. O'Connell NE, Marston L, Spencer S, DeSouza LH, Wands BM. Non-invasive brain stimulation techniques for chronic pain. Cochrane Db Syst Rev. 2018(3).
6. Saltychev M, Laimi K. Effectiveness of repetitive transcranial magnetic stimulation in patients with fibromyalgia: a meta-analysis. Int J Rehabil Res. 2017;40(1):11-8.

For peer review only - http://bmjopen.bmj.com/site/about/guidelines.xhtml

7. Yang SY, Chang MC. Effect of Repetitive Transcranial Magnetic Stimulation on Pain Management: A Systematic Narrative Review. Frontiers in Neurology. 2020;11.
8. Lefaucheur JP, Aleman A, Baeken C, Benninger DH, Brunelin J, Di Lazzaro V, et al. Evidence-based guidelines on the therapeutic use of repetitive transcranial magnetic stimulation (rTMS): An update (2014-2018). Clin Neurophysiol. 2020;131(2):474-528.
9. Galhardoni R, Correia GS, Araujo H, Yeng LT, Fernandes DT, Kaziyama HH, et al. Repetitive Transcranial Magnetic Stimulation in Chronic Pain: A Review of the Literature. Archives of physical medicine and rehabilitation. 2015;96(4):S156-S72.
10. Pszczolkowski S, Cottam WJ, Briley PM, Iwabuchi SJ, Kaylor-Hughes C, Shalabi A, et al. Connectivity-Guided Theta Burst Transcranial Magnetic Stimulation Versus Repetitive Transcranial Magnetic Stimulation for Treatment-Resistant Moderate to Severe Depression: Magnetic Resonance Imaging Protocol and SARS-CoV-2-Induced Changes for a Randomized Double-blind Controlled Trial. JMIR Res Protoc. 2022;11(1).
11. Morriss R, Webster L, Abdelghani M, Auer DP, Barber S, Bates P, et al. Connectivity guided theta burst transcranial magnetic stimulation versus repetitive transcranial magnetic stimulation for treatment-resistant moderate to severe depression: study protocol for a randomised double-blind controlled trial (BRIGhTMIND). BMJ Open. 2020;10(7).
12. Blumberger DM, Vila-Rodriguez F, Thorpe KE. Effectiveness of theta burst versus high-frequency repetitive transcranial magnetic stimulation in patients with depression (THREE-D): a randomised non-inferiority trial (vol 391, pg 1683, 2018). Lancet. 2018;391(10139):E24-E.
13. Fitzgerald PB. An update on the clinical use of repetitive transcranial magnetic stimulation in the treatment of depression. J Affect Disord. 2020;276:90-103.
14. Duprat R, Desmyter S, De Raedt R, van Heeringen K, Van den Abbeele D, Tandt H, et al. Accelerated intermittent theta burst stimulation treatment in medication-resistant major depression: A fast road to remission? J Affect Disord. 2016;200:6-14.
15. Cole EJ, Stimpson KH, Bentzley BS, Gulser M, Cherian K, Tischler C, et al. Stanford Accelerated Intelligent Neuromodulation Therapy for Treatment-Resistant Depression. Am J Psychiat. 2020;177(8):716-26.
16. Fox MD, Buckner RL, White MP, Greicius MD, Pascual-Leone A. Efficacy of Transcranial Magnetic Stimulation Targets for Depression Is Related to Intrinsic Functional Connectivity with the Subgenual Cingulate. Biol Psychiat. 2012;72(7):595-603.
17. Kessler S, Grammozis A, Gunther KP, Kirschner S. The Intermittent and Constant Pain Score (ICOAP) - a Questionnaire to Assess Pain in Patients with Gonarthritis. Z Orthop Unfallchir. 2011;149(1):22-6.
18. Akin-Akinyosoye K, James RJE, McWilliams DF, Millar B, das Nair R, Ferguson E, et al. The Central Aspects of Pain in the Knee (CAP-Knee) questionnaire; a mixed-methods study of a self-report instrument for assessing central mechanisms in people with knee pain. Osteoarthr Cartilage. 2021;29(6):802-14.
19. Freynhagen R, Baron R, Gockel U, Tolle TR. painDETECT: a new screening questionnaire to identify neuropathic components in patients with back pain. Curr Med Res Opin. 2006;22(10):1911-20.
20. Sullivan MJL, Bishop SR, Pivik J. The Pain Catastrophizing Scale: Development and validation. Psychol Assessment. 1995;7(4):524-32.
21. Williams N. The GAD-7 questionnaire. Occup Med-Oxford. 2014;64(3):224-.
22. Henry JD, Crawford JR. The short-form version of the Depression Anxiety Stress Scales (DASS-21): construct validity and normative data in a large non-clinical sample. Br J Clin Psychol. 2005;44(Pt 2):227-39.
23. Kenn C, Wood H, Kucyj M, Wattis J, Cunane J. Validation of the Hospital Anxiety and Depression Rating-Scale (Hads) in an Elderly Psychiatric Population. Int J Geriatr Psych. 1987;2(3):189-93.
24. Lowe B, Kroenke K, Herzog W, Grafe K. Measuring depression outcome with a brief self-report instrument: sensitivity to change of the Patient Health Questionnaire (PHQ-9). J Affect Disord. 2004;81(1):61-6.

For peer review only - http://bmjopen.bmj.com/site/about/guidelines.xhtml

25. Nasreddine ZS, Chertkow H, Phillips N, Whitehead V, Collin I, Cummings JL. The Montreal Cognitive Assessment (MoCA): a brief cognitive screening tool for detection of Mild Cognitive Impairment. Neurology. 2004;62(7):A132-A.
26. Whitehead AL, Julious SA, Cooper CL, Campbell MJ. Estimating the sample size for a pilot randomised trial to minimise the overall trial sample size for the external pilot and main trial for a continuous outcome variable. Stat Methods Med Res. 2016;25(3):1057-73.

For peer review only

For peer review only - http://bmjopen.bmj.com/site/about/guidelines.xhtml

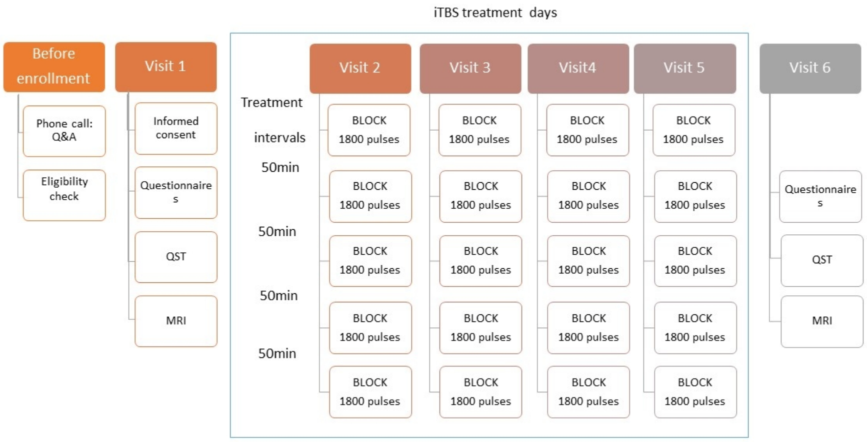

Figure 1: Pilot study overview

338x190mm (96 x 96 DPI)

STANDARD PROTOCOL ITEMS: RECOMMENDATIONS FOR INTERVENTIONAL TRIALS

SPIRIT 2013 Checklist for the ReTrain pilot RCT: Recommended items to address in a clinical trial protocol and related documents*

Section/item	Item No	Description	Addressed on page number
**Administrative information**			
Title	1	Descriptive title identifying the study design, population, interventions, and, if applicable, trial acronym	1
Trial registration	2a	Trial identifier and registry name. If not yet registered, name of intended registry	2
	2b	All items from the World Health Organization Trial Registration Data Set	See clinical trial registration
Protocol version	3	Date and version identifier	1
Funding	4	Sources and types of financial, material, and other support	15
Roles and responsibilities	5a	Names, affiliations, and roles of protocol contributors	1, 15
	5b	Name and contact information for the trial sponsor	1
	5c	Role of study sponsor and funders, if any, in study design; collection, management, analysis, and interpretation of data; writing of the report; and the decision to submit the report for publication, including whether they will have ultimate authority over any of these activities	15
	5d	Composition, roles, and responsibilities of the coordinating centre, steering committee, endpoint adjudication committee, data management team, and other individuals or groups overseeing the trial, if applicable (see Item 21a for data monitoring committee)	15,14,12,10

For peer review only

**Introduction**			
Background and rationale	6a	Description of research question and justification for undertaking the trial, including summary of relevant studies (published and unpublished) examining benefits and harms for each intervention	3- 5
	6b	Explanation for choice of comparators	10,11
Objectives	7	Specific objectives or hypotheses	6
Trial design	8	Description of trial design including type of trial (eg, parallel group, crossover, factorial, single group), allocation ratio, and framework (eg, superiority, equivalence, noninferiority, exploratory)	5
**Methods: Participants, interventions, and outcomes**			
Study setting	9	Description of study settings (eg, community clinic, academic hospital) and list of countries where data will be collected. Reference to where list of study sites can be obtained	5
Eligibility criteria	10	Inclusion and exclusion criteria for participants. If applicable, eligibility criteria for study centres and individuals who will perform the interventions (eg, surgeons, psychotherapists)	7,8
Interventions	11a	Interventions for each group with sufficient detail to allow replication, including how and when they will be administered	8-11
	11b	Criteria for discontinuing or modifying allocated interventions for a given trial participant (eg, drug dose change in response to harms, participant request, or improving/worsening disease)	12,13
	11c	Strategies to improve adherence to intervention protocols, and any procedures for monitoring adherence (eg, drug tablet return, laboratory tests)	13
	11d	Relevant concomitant care and interventions that are permitted or prohibited during the trial	11-13
Outcomes	12	Primary, secondary, and other outcomes, including the specific measurement variable (eg, systolic blood pressure), analysis metric (eg, change from baseline, final value, time to event), method of aggregation (eg, median, proportion), and time point for each outcome. Explanation of the clinical relevance of chosen efficacy and harm outcomes is strongly recommended	11-13
Participant timeline	13	Time schedule of enrolment, interventions (including any run-ins and washouts), assessments, and visits for participants. A schematic diagram is highly recommended (see Figure)	6

For peer review only - http://bmjopen.bmj.com/site/about/guidelines.xhtml

Sample size	14	Estimated number of participants needed to achieve study objectives and how it was determined, including clinical and statistical assumptions supporting any sample size calculations	13,14
Recruitment	15	Strategies for achieving adequate participant enrolment to reach target sample size	7

Methods: Assignment of interventions (for controlled trials)

Allocation:

Sequence generation	16a	Method of generating the allocation sequence (eg, computer-generated random numbers), and list of any factors for stratification. To reduce predictability of a random sequence, details of any planned restriction (eg, blocking) should be provided in a separate document that is unavailable to those who enrol participants or assign interventions	10
Allocation concealment mechanism	16b	Mechanism of implementing the allocation sequence (eg, central telephone; sequentially numbered, opaque, sealed envelopes), describing any steps to conceal the sequence until interventions are assigned	10
Implementation	16c	Who will generate the allocation sequence, who will enrol participants, and who will assign participants to interventions	10
Blinding (masking)	17a	Who will be blinded after assignment to interventions (eg, trial participants, care providers, outcome assessors, data analysts), and how	10
	17b	If blinded, circumstances under which unblinding is permissible, and procedure for revealing a participant's allocated intervention during the trial	10

Methods: Data collection, management, and analysis

Data collection methods	18a	Plans for assessment and collection of outcome, baseline, and other trial data, including any related processes to promote data quality (eg, duplicate measurements, training of assessors) and a description of study instruments (eg, questionnaires, laboratory tests) along with their reliability and validity, if known. Reference to where data collection forms can be found, if not in the protocol	14
	18b	Plans to promote participant retention and complete follow-up, including list of any outcome data to be collected for participants who discontinue or deviate from intervention protocols	n.a.

Data management	19	Plans for data entry, coding, security, and storage, including any related processes to promote data quality (eg, double data entry; range checks for data values). Reference to where details of data management procedures can be found, if not in the protocol	10
Statistical methods	20a	Statistical methods for analysing primary and secondary outcomes. Reference to where other details of the statistical analysis plan can be found, if not in the protocol	14
	20b	Methods for any additional analyses (eg, subgroup and adjusted analyses)	n.a.
	20c	Definition of analysis population relating to protocol non-adherence (eg, as randomised analysis), and any statistical methods to handle missing data (eg, multiple imputation)	n.a.
**Methods: Monitoring**			
Data monitoring	21a	Composition of data monitoring committee (DMC); summary of its role and reporting structure; statement of whether it is independent from the sponsor and competing interests; and reference to where further details about its charter can be found, if not in the protocol. Alternatively, an explanation of why a DMC is not needed	14
	21b	Description of any interim analyses and stopping guidelines, including who will have access to these interim results and make the final decision to terminate the trial	12
Harms	22	Plans for collecting, assessing, reporting, and managing solicited and spontaneously reported adverse events and other unintended effects of trial interventions or trial conduct	13
Auditing	23	Frequency and procedures for auditing trial conduct, if any, and whether the process will be independent from investigators and the sponsor	n.a.
**Ethics and dissemination**			
Research ethics approval	24	Plans for seeking research ethics committee/institutional review board (REC/IRB) approval	1
Protocol amendments	25	Plans for communicating important protocol modifications (eg, changes to eligibility criteria, outcomes, analyses) to relevant parties (eg, investigators, REC/IRBs, trial participants, trial registries, journals, regulators)	14

Consent or assent	26a	Who will obtain informed consent or assent from potential trial participants or authorised surrogates, and how (see Item 32)	6
	26b	Additional consent provisions for collection and use of participant data and biological specimens in ancillary studies, if applicable	n.a.
Confidentiality	27	How personal information about potential and enrolled participants will be collected, shared, and maintained in order to protect confidentiality before, during, and after the trial	See participant information sheet
Declaration of interests	28	Financial and other competing interests for principal investigators for the overall trial and each study site	15
Access to data	29	Statement of who will have access to the final trial dataset, and disclosure of contractual agreements that limit such access for investigators	See clinical trial registry
Ancillary and post-trial care	30	Provisions, if any, for ancillary and post-trial care, and for compensation to those who suffer harm from trial participation	See participant information sheet.
Dissemination policy	31a	Plans for investigators and sponsor to communicate trial results to participants, healthcare professionals, the public, and other relevant groups (eg, via publication, reporting in results databases, or other data sharing arrangements), including any publication restrictions	14
	31b	Authorship eligibility guidelines and any intended use of professional writers	15
	31c	Plans, if any, for granting public access to the full protocol, participant-level dataset, and statistical code	n.a.
**Appendices**			
Informed consent materials	32	Model consent form and other related documentation given to participants and authorised surrogates	Attached.
Biological specimens	33	Plans for collection, laboratory evaluation, and storage of biological specimens for genetic or molecular analysis in the current trial and for future use in ancillary studies, if applicable	n.a.

*It is strongly recommended that this checklist be read in conjunction with the SPIRIT 2013 Explanation & Elaboration for important clarification on the items. Amendments to the protocol should be tracked and dated. The SPIRIT checklist is copyrighted by the SPIRIT Group under the Creative Commons "Attribution-NonCommercial-NoDerivs 3.0 Unported" license.

BMJ Open

Brain connectivity-guided, optimised theta burst transcranial magnetic stimulation to improve central pain modulation in knee osteoarthritis pain (BoostCPM): protocol of a pilot randomised clinical trial in a secondary care setting in the UK.

Journal:	*BMJ Open*
Manuscript ID	bmjopen-2023-073378.R2
Article Type:	Protocol
Date Submitted by the Author:	13-Sep-2023
Complete List of Authors:	Drabek, Marianne; University of Nottingham,<br>Hodkinson, Duncan; Nottingham NIHR Biomedical Research Centre; University of Nottingham<br>Horvath, Suzanne; Nottingham NIHR Biomedical Research Centre<br>Millar, Bonnie; University of Nottingham Faculty of Medicine and Health Sciences, Academic Rheumatology; NIHR Nottigham Biomedical Research Centre, Academic Rheumatology<br>Pszczolkowski Parraguez, Stefan; University of Nottingham<br>Tench, Christopher; University of Nottingham, Division of Clinical Neuroscience<br>Tanasescu, Radu; University of Nottingham,<br>Lankappa, Sudheer; Nottinghamshire Healthcare NHS Foundation Trust, Adult Mental Health<br>Morriss, Richard; University of Nottingham, Psychiatry<br>Walsh, David; University of Nottingham, Academic Rheumatology<br>Auer, Dorothee; Nottingham NIHR Biomedical Research Centre; University of Nottingham
**Primary Subject Heading**:	Pharmacology and therapeutics
Secondary Subject Heading:	Rheumatology, Mental health, Neurology
Keywords:	Chronic Pain, PAIN MANAGEMENT, Magnetic Resonance Imaging, Randomized Controlled Trial, Transcranial Magnetic Stimulation

SCHOLARONE™
Manuscripts

Brain connectivity-guided, **o**ptimised theta burst transcranial magnetic stimulation to improve **c**entral **p**ain **m**odulation in knee **o**steoarthritis **p**ain (BoostCPM): protocol of a pilot randomised clinical trial in a secondary care setting in the UK.

Authors

Marianne M. Drabek[1,2,3], Duncan Hodkinson[1,2,3], Suzanne Horvath[1], Bonnie Millar[1,3], Stefan Pszczolkowski[1,2], Christopher Tench[1,2, 5], Radu Tanasescu[1,4,5], Sudheer T Lankappa[6], Richard Morriss[1], David A. Walsh[3], Dorothee P. Auer[1,2,3]

Affiliations

[1] Nottingham NIHR Biomedical Research Centre

[2] Sir Peter Mansfield Imaging Centre, School of Medicine, University of Nottingham, Nottingham

[3] Pain Centre Versus Arthritis, University of Nottingham, Nottingham

[4] Department of Neurology, Queen's Medical Centre, Nottingham University Hospital Trust

[5]Clinical Neurology, School of Medicine, University of Nottingham

[6]Psychiatry, Nottinghamshire Healthcare Foundation Trust

Corresponding author

Dorothee P. Auer, Radiological Sciences, Room W/B 1441, Queen's Medical Centre, Nottingham, NG7 2UH, UK. Email: dorothee.auer@nottingham.ac.uk

Word count: 3979 (Introduction-Dissemination inclusive)

Key words: Transcranial Magnetic Stimulation, chronic pain, refractory pain, osteoarthritis, brain connectivity, dorsolateral prefrontal cortex

For peer review only

Abstract

Introduction

Chronic pain is a common health problem that is not efficiently managed by standard analgesic treatments. There is evidence that treatment resistance may result from maladaptive brain changes in areas that are fundamental to the perception of pain. Knee osteoarthritis is one of the most prevalent causes of chronic pain and commonly associated with negative affect. Chronic knee osteoarthritis pain is also associated with altered right anterior insula functional connectivity. We posit that reversal of these brain circuit alterations may be critical to alleviate chronic pain and associated negative affect, and that this can be achieved through non-invasive neuromodulation techniques. Despite growing interest in non-invasive neuromodulation for pain relief and proven efficacy in depression, results in chronic pain are mixed with limited high-quality evidence for clinical and mechanistic efficacy. Limitations include patient heterogeneity, imprecision of target selection, uncertain blinding and protocols that may deliver pulses at subclinical efficacy.

Methods and Analysis

We hence developed an optimised treatment protocol of connectivity-guided intermittent theta-burst stimulation (iTBS) targeting the left dorso-lateral prefrontal cortex with accelerated delivery on four consecutive days (allowing four days within the same week as protocol variation) with 5 daily treatment sessions that will be piloted in a sham-controlled design in 45 participants with chronic knee pain. This pilot study protocol will assess feasibility, tolerability and explore mechanistic efficacy through serial functional/structural MRI and quantitative sensory testing.

Ethics and Dissemination

This pilot trial has been approved by the Ethics Committee Cornwall & Plymouth.
Results of the pilot trial will be submitted to peer-reviewed journals, presented at research conferences and may be shared with participants and PPI/E advisors.

Trial registration

The trial has been registered (Clinical Trial Reference: ISRCTN15404076)

For peer review only - http://bmjopen.bmj.com/site/about/guidelines.xhtml

Strengths and limitations of this study

- The protocol combines connectivity-guided neuronavigation for improved precision to target the prefronto-insular circuit with accelerated delivery of five daily iTBS sessions over four days in one week for enhanced efficacy and efficiency in a sham-controlled RCT design.
- Advanced serial MRI of brain network function complements psychophysical and questionnaire outcome markers before and after TMS protocol to explore mechanistic efficacy.
- Recruitment is predominantly from a research-ready community cohort which together with the single centre design may limit generalisability.
- The intense protocol may only be accommodated and tolerated by a subgroup of pain patients.
- The total number of 36,000 pulses and the permitted reduction of RMT below 80% may limit efficacy of the intervention.

For peer review only

Introduction

Chronic pain is a major burden for society, economy, health systems and individuals. There is a lack of effective analgesic treatments in chronic pain with an urgent need for novel treatments. Maladaptive brain mechanisms are increasingly recognised as key contributors to the development and persistence of pain (1). A feature is the altered communication between brain areas that are involved in the processing of pain signals and the modulation of the associated pain perception, demonstrated by dysfunctional connectivity of the right anterior insula (2).

These maladaptive functional brain network changes are thought (i) to drive the experience of chronic pain and central pain sensitisation, (ii) to be potentially reversible by non-invasive neuromodulation, and thus support the notion that interventions that normalise the brain's (and in particular fronto-limbic) connectivity will also reverse central pain augmentation or associated affective experience.

Repetitive transcranial Magnetic Stimulation (rTMS) is a promising tool to non-invasively and focally stimulate the brain through induction of small electrical currents. Choice of stimulation protocols are thought to determine excitatory or inhibitory local and downstream effects with acute and longer-term neuromodulatory effects on targeted brain circuits. The exact mechanism of acute neuromodulatory and neuroplastic effects and the substantial inter-and intraindividual variability are poorly understood (3). Clinical efficacy of rTMS is well established for the treatment of major depression, approved by the Federal Drug Administration (FDA) in the US and the National Institute

For peer review only - http://bmjopen.bmj.com/site/about/guidelines.xhtml

of Clinical Excellence (NICE) in the UK. In chronic pain, to date evidence for effective neuromodulation is limited to invasive protocols and selected indications (4). There is however considerable recent interest in non-invasive brain stimulation (NIBS) trials in chronic pain yet results to date are mixed. Meta-analyses and systematic reviews have described NIBS as having 'poor or inconclusive' analgesic effects across sites and pain disorders (4-6), "minimally clinically relevant"(6), to "promising"(7) and "probable efficacy" for either motor cortex (M1) or dorsolateral prefrontal cortex (DLPFC) stimulation (8). There are major quality concerns with most studies considered poor (4) due to underpowered sample sizes, lack of blinding, inadequate randomization, or missing sham for comparisons. Moreover, there are uncertainties regarding the most effective brain stimulation site, type of stimulation, target population. Nevertheless, pain relief was frequently found to be >30% (9) which encourages to seek further improvements and address limitations in order to fully assess and exploit the potential that this technology may hold for pain treatment.

To address these shortcomings we chose to target the left DLPFC as a particularly promising yet relatively understudied target site for rTMS in chronic pain due to its strong modulatory influence on the right anterior insula (rAI), a key hub in pain processing with known altered connectivity in chronic pain (2). Stimulating the left DLPFC is expected to modulate the prefronto-insular pathway as a putative central pain modulatory effect. This pathway is also a key target in the clinically effective rTMS treatment of major depression suggesting a second putative beneficial effect through reduction of negative affect and related pain augmentation.

rsfMRI based effective connectivity to determine the coordinates within the anatomically defined left DLPFC that show the strongest effective connectivity to the right anterior insula as in our previous work (10,11).

In addition, recently accelerated, optimised TMS protocols were reported to be safe and similarly effective to standard rTMS using intermittent theta burst stimulation (iTBS) (12-15). There are several advantages expected from iTBS protocols that are faster than standard rTMS and may have stronger neuroplasticity effects. Also, the multiple treatment blocks per day with 50 minutes intervals are expected to further enhance effectiveness and allow to deliver a higher cumulative weekly dose with the benefit of shorter overall treatment course.

Motivated by the promising results of the optimised iTBS protocol in major depression, we translated and adapted the approach to chronic musculoskeletal pain, which, if effective, would greatly improve clinical interest in this technique both for cost and practical reasons. As study population we chose knee osteoarthritis (OA) as one of the most common causes of chronic musculoskeletal pain and to

For peer review only - http://bmjopen.bmj.com/site/about/guidelines.xhtml

avoid potential heterogeneity from different pain aetiology. Based on the known comorbidity, we anticipate that the study population of moderate chronic knee OA pain will report higher negative affect than the general population which may further increase the likelihood of neuromodulatory efficacy at the chosen target site.

This parallel, sham-controlled pilot study will investigate a four day (over one week) intense, imaging-guided intermittent theta burst stimulation protocol that is individually targeted within the left DLPFC to maximise the connectivity with the rAI. Study participants with moderately severe, chronic knee pain will undergo serial MRI, behavioural and quantitative sensory testing before and after the iTBS/sham intervention and online recording of patient-reported outcomes until week 18 to guide the protocol and sample size for future RCT interventions.

Objectives

(i) To test the feasibility and tolerability of an optimis ed accelerated four day (over one week) iTBS protocol with 5 daily sessions (total pulses 36,000 aimed at 80% RMT) using connectivity-guided targeting of the left DLPFC in chronic knee OA patients with moderately severe pain.

(ii) To assess the variance of mechanistic outcome markers comparing the intense iTBS protocol versus sham.

(iii) To explore clinical efficacy metrics, and the feasibility of online recorded patient-reported outcome measures in the iTBS and sham arm.

(iv) To explore the effects of iTBS on negative affect, stress and arousal between iTBS and sham.

(v) To assess feasibility of blinding using sham TMS.

Methods and Analysis

Trial design

Single centre, sham controlled randomised mechanistic pilot study at the University of Nottingham with a parallel group study design, whereby participants will either receive active iTBS or sham iTBS with random allocation of 2:1. The uneven allocation was chosen to achieve blinding but preferencing power for statistical analyses in the active condition.

Hypotheses

Primary mechanistic hypotheses

For peer review only - http://bmjopen.bmj.com/site/about/guidelines.xhtml

1. Accelerated, personalised iTBS of the DLPFC modulates *functional activity and connectivity* of brain circuits involved in central pain control in chronic musculoskeletal pain compared with sham controls. The direction of change is expected to reduce hallmark features of the pain connectome, such as reduction of the abnormally increased rAI-posterior default mode network anticorrelation.
2. Accelerated, personalised iTBS of the DLPFC increases endogenous antinociception in chronic MSK pain compared to sham controls.

Secondary Hypotheses

3. Accelerated, personalised iTBS of the DLPFC reduces pain catastrophizing, anxiety and improves mood in chronic musculoskeletal pain compared to sham controls.
4. Accelerated, personalised iTBS of the DLPFC reduces sensitivity to experimental pain at the end of the intervention compared to sham controls.
5. Accelerated, personalised iTBS of the DLPFC reduces physiological arousal and reported stress levels compared to sham controls.

Study Procedures

Participants will first be assessed over phone for meeting safety and eligibility criteria before giving informed written consent. Participants will be asked to attend for baseline assessments (visit 1) (ca. 2 hours including MRI, and psycho-physical assessment including brief experimental pain exposure) (see Figure 1). Then participants will undergo an intervention week with four visits (Visit 2-5) lasting about half a day each. Each visit will consist of five blocks of iTBS or sham with inter-block interval of ~50 minutes during which participants will be offered refreshments and food in a comfortable waiting area before getting prepared for the next session. The 6th and final study visit is a repeat of the baseline visit, and will be scheduled within 18 -72 hours of the last treatment session. This will be followed by online patient self-recording of pain and emotional outcomes up to 16 weeks.

Recruitment and inclusion criteria

Participants will be recruited primarily from previous participants who consented to be contacted (such as the community-based Investigating Musculoskeletal Health and Wellbeing cohort) (16) and additional recruitment pathways via posters, general practitioner surgeries and other NHS services (Joint replacement waiting list) or co-recruited from ongoing studies that allow co-recruitment as required. Individuals who consented to be contacted about new studies, will be contacted by the study

For peer review only - http://bmjopen.bmj.com/site/about/guidelines.xhtml

team who originally consented the participant; only if the participant then expresses interest in the new study and agrees for their contact details to be passed on to the new study team, the latter will contact them.

Participants may be initially invited to take part in the study by sending a short format participant information sheet (PIS) form that summarizes the main points of the study for consideration of interest; participants who contact for further information will be sent the final PIS. Alternatively, they may find the short format PIS as a poster and contact us. Posters may be put around the Nottingham University Hospital Trust, the Medical School and other University of Nottingham sites, public sites such as public libraries and leisure/community centres. Posters may also be placed on local press, including but not limited to The Nottingham Post and Metro. The same advert may be featured on UK websites related to chronic pain, including but not limited to the Pain Centre Versus Arthritis webpages within http://www.nottingham.ac.uk/paincentre.

Participant enrolment and retention will be continually monitored, and numbers fed back at the study team meetings. Feedback from participants will be used to maintain recruitment and retention rates.

Inclusion criteria

- 18-75 years of age and able to consent
- Chronic knee pain (minimum 6 months duration) with VAS score >=4/10
- Able to accommodate the study visits without disruption to their jobs or other responsibilities and without experiencing excessive physical strain or other distress.

Exclusion criteria

Patients are admitted to the study if they also meet the following criteria:

- No contraindication to MRI and TMS
- No major medical or neurological conditions unrelated to the pain condition.
- Not scheduled for total knee replacement surgery (TKR) within 1 month of study visits or other considerable treatment change
- No change in pain medication in the past four weeks,
- Not on centrally active medication other than stable antidepressants or on opioidergic analgesic treatment.
- Not on medication, alcohol or recreational drugs, thought to increase risk of seizure and /or syncope.
- Not experiencing frequent headaches.

For peer review only - http://bmjopen.bmj.com/site/about/guidelines.xhtml

Participants will be reimbursed for travel expenses and provided with free drinks and food during study sessions but will not be compensated for their time.

Transcranial magnetic stimulation

Transcranial magnetic stimulation (TMS) will be delivered using the Magstim Horizon Performance system with StimGuide Navigated TMS package (The Magstim Co. Ltd). Single-pulse TMS will be applied to the left motor cortex using a handheld figure-of-eight coil (diameter 7cm) to determine the resting motor threshold (RMT). RMT is defined as the minimum stimulator output intensity needed to achieve a minimum motor evoked potential (MEP)-amplitude of 50μV in the completely relaxed abductor pollicis brevis (APB)-muscle in at least 5 out of 10 trials. For all treatments, the intermittent theta burst stimulation (iTBS) protocol will be delivered using a fixed 7cm diameter coil (E-z Cool Coil and support arm) consisting of 3 pulses (aimed at 80% RMT, but may be lowered to avoid participant discomfort) of 50Hz repeated at 200ms (5Hz) intervals with a total 1800 pulses per block, 9000 pulses per visit/day and 36,000 pulses in total/ treatment week.

The staff operating the TMS device (SZ, senior research nurse with 3 years' experience in rTMS treatment/trials; DH senior research fellow with 2 years' experience in research TMS) are trained on the MAGSTIM system and adhere to all safety instructions.

MRI acquisition

Imaging data will be acquired on a GE Premier 3T MR scanner using a 48-channel head coil (GE Healthcare, USA). Structural scans include high resolution T1-weighted (T1w) and T2-weighted (T2w) images for the purpose of image registration. The T1w images will be acquired using 3D MPRAGE sequence (voxel size=1mm isotropic, FOV = 256, matrix = 256, 256 sagittal slices, TI = 800 ms, FA = 8°) and T2w will be images acquired using 3D Cube T2 FLAIR sequence (voxel size=1mm isotropic, FOV = 256, matrix = 256, 256 sagittal slices, TR = 6300ms, TE =121ms, TI =1787ms). RsfMRI data will be acquired using whole-brain 2D GE-EPI sequence (TR=1400ms, TE=35ms, flip angle=68°, in-plane FOV = 212×212mm, 57 slices, slice thickness=2mm, voxel size=2mm isotropic, hyperband factor=3, ARC factor=2, 15min total scan time). To measure the b_0 field for correction of EPI distortions, we will also acquire two SE-EPI images with reversed phase encoding directions. The SE-EPI images have the same geometry, echo spacing, and phase encoding direction parameters as the GE fMRI scans.

Image processing and TMS neuronavigation

For peer review only - http://bmjopen.bmj.com/site/about/guidelines.xhtml

Image pre-processing follows the same procedure to that described in Pszczolkowski et al. (10). In brief, the structural T1-weighted images are brain extracted, bias corrected and segmented into white matter (WM), grey matter (GM) and cerebrospinal fluid (CSF) probability maps which are binarized at a probability of 0.98 and then eroded using a spherical kernel with a radius of 2 voxels to obtain the final tissue labels. The blood oxygenation level dependent (BOLD) rsfMRI undergo EPI distortion correction, motion correction, slice timing correction, smoothing, denoising and high-pass filtering. Finally, the WM and GM tissue labels are transformed into rsfMRI BOLD space and used to regress out the WM and GM time series from the processed rsfMRI BOLD data.

For identification of the iTBS treatment target, a methodology similar to Pszczolkowski et al. (10) will be followed. However, in the current pilot trial the DLPFC searchlight described in Fox et al. (17) will be used as primary volume of interest, and the background removal procedure is based on a threshold computed automatically rather than by visual inspection. This threshold is computed as the 98th percentile of the T1-weighted intensities within a mask of background (air) voxels around the outside of the skull in MNI space which is previously transformed into T1-weighted space.

Quantitative sensory testing (QST)

Central pain inhibition will be tested pre- and post-TBS treatment using the psychophysical paradigm of conditioned pain modulation (CPM). The test stimulus will be pressure pain threshold (PPT) and the conditioning stimulus ischemic pain caused by a blood pressure cuff placed at the calf of the unaffected leg. The test stimulus (PPTs) will be administered at three body sites (on the affected side), including trapezius muscle, metacarpophalageal joint of the thumb, and the patella. If no preference is shown with respect to the affected side, then the patients non-dominant body side will be examined. All QST assessment are completed by DH with > 10 years' experience.

Conditioned pain modulation is calculated as follows:

CPM (%) = (PPTpost-conditioning – PPTpre-conditioning)/ PPTpre-conditioning X 100.

Pre-conditioning PPTs will be used to calculate SEM and >±2XSEM to determine CPM effects in response TBS treatment.

Sham, randomisation and blinding

The sham intervention will be given through the same coil as the iTBS treatment and both active and sham will be administered over the left DLPFC using neuronavigation co-ordinates (s. above). For the

For peer review only - http://bmjopen.bmj.com/site/about/guidelines.xhtml

sham group, pulses will be delivered at 30% stimulator output by orienting the coil perpendicular to the scalp surface with direct contact. This will ensure that an appropriate audible noise is created to reinforce the integrity of the treatment blinding.

The study statistician will generate allocation by randomising blocks of participants, ensuring age and sex matching between arms. Block randomisation (block size 6) will be used determined using online tools (http://www.jerrydallal.com/random/randomize.htm). Initially 30 subjects will be randomised with 3 subjects per group in each block. Given the recruitment rates and feasibility of recruiting all subjects within the time frame, the next subjects will be randomised similarly. However, if recruitment suggests a shortfall of subjects within the time frame the block allocation will be adjusted such that a higher rate of subjects are recruited to the treatment group such as to ensure sufficient numbers in that group. This is expected to be at most a small adjustment such that the block of 6 will still be sufficient to effectively mask assignment, for example 4 into the treatment group and 2 into the sham group. The study co-ordinator (BM) will enrol and assign participants.

Study participants will be blinded to the intervention allocation and participants will be asked whether they can guess the type of intervention as part of an TMS exist interview/questionnaire. The study team involved in delivering the intervention cannot be blinded, but the data analysis team will be blinded to which intervention was delivered. Participants will be unblinded if they were to experience adverse events.

Assessments

Data will be collected for the following

- MRI at visit 1 and at visit 6 (pre- and post-treatment)
- Quantitative sensory testing (QST) at visit 1 and at visit 6 (pre- and post-treatment)

RedCap (https://www.project-redcap.org/) will be used to administer online questionnaires:

Pain characteristics

- a numerical pain VAS (0-10) as online daily pain report
- weekly questionnaires:
  Intermittent and Constant Pain Score (ICOAP) (18), The Central Aspects of Pain in the Knee (CAP-Knee) questionnaire (19)
- at visit 1 &6:

For peer review only - http://bmjopen.bmj.com/site/about/guidelines.xhtml

painDETECT (20), Pain Catastrophizing Scale (PCS)(21), ICOAP (18), General Anxiety Depression scale (GAD-7)(22), Depression Anxiety Stress Scales (DASS-21)(23), Hospital Anxiety and Depression Rating-Scale (HADS)(24), Patient Health Questionnaire (PHQ-9)(25), CAP-Knee (19), Montreal Cognitive Assessment (MoCA) Test (26).

Outcome metrics and measures

Outcome metrics are changes between end of the intervention (Visit 6) and baseline (Visit 1) of optimized neuroimaging markers of the DPMS that will be established from independent datasets and pre-defined in the analysis plan and secondly markers of central pain modulation, derived from QST.

Outcome measures

Primary:

1. Variance of changes in pre-selected neuroimaging functional markers of the descending pain modulatory system at final vs baseline visit.
2. Variance of change in conditioned pain modulation (pain intensity change with / without conditioned pain stimulus) at final vs baseline visit.
3. Number of participants recruited and retained on the trial.
4. Number of iTBS sessions completed at 80% RMT.

Secondary:

5. Variance of change in reported pain intensity, burden and catastrophizing at final versus baseline study visit (ICOAP, PCS)
6. Change in negative affect (HADS) at final versus baseline study visit.
7. Variance in physiological and psychophysical changes (acute experimental pain) at final vs baseline study visit.
8. Variance of PROMS (daily online recording pain, wellbeing, sleep) over 16 weeks from treatment versus enrolment-baseline.

Adherence, tolerability, withdrawal and pausing of study trial

Adherence in this study will be measured by participants' attendance at study visits, completion of all study measures and procedures, and TMS treatments. We will also record the tolerability of 80% RMT and any necessary adjustments of %RMT and NIBS stimulation due to discomfort. Safety instructions

For peer review only - http://bmjopen.bmj.com/site/about/guidelines.xhtml

For peer review only

also include not being intoxicated by alcohol or non-prescription drugs at any study visit, and any history of heavy binges of alcohol or intermittent hypnotic or benzodiazepine use in the 24 hours before a treatment session because of these behaviours would increase the risk of seizures or syncope with TMS.

Participants who fail to comply with safety instructions can be withdrawn from the study at any time. Study withdrawal could be requested anytime by the participants.

Tolerability of the accelerated protocol will be individually monitored in the first 10 participants with planned meeting of the study team with the independent medical safety advisor (RT) for go/no-go decision making should the study retention exceed expected levels for rTMS studies or based on concerning individual feedback. Within the first 10 participants we did not observe withdrawal due to protocol non-compliance.

Known Side Effects and Discomfort

The staff operating the TMS device (S 3years, DH 2 years) are trained and adhere to all safety instructions.

As it directly influences activity of nerve cells, theoretically there is the risk of a seizure although the risk level is considered low. The cumulative number of TMS pulses is 36,000 which is substantially lower than the accelerated Stanford protocol and lower than the FDA approved protocol (48,000-72,000 for 4-6 weeks).

Staff and participants will be wearing ear protectors because of the risk of tinnitus and hearing impairment from the loudness of the TMS stimulus.

The majority of TMS treatments are very well tolerated, occasionally however a short-lasting headache can occur. Depending on an individual’s facial anatomy, facial nerves can be stimulated at the same time which would result in muscle twitches in the face; these however cease as the stimulation is stopped.

Should a participant experience any side effects and find it too uncomfortable, the treatment session can be stopped immediately but where possible, adjustments will be made to ease discomfort. To reduce participants' discomfort on the first day of the session, the power was individually ramped to 80% RMT or lower depending on subjective tolerability.

For peer review only - http://bmjopen.bmj.com/site/about/guidelines.xhtml

A standard operating procedure has been developed that defines the discontinuation of the trial intervention, referral to treatment as required and risk management of the most common serious side effect (seizure) and more common side effects like headache and dizziness.

Safety assessment

Despite the safety report on accelerated iTBS protocols, side-effects and tolerability in a chronic pain population were unknown, hence the trial will be monitored with the intention to stop should a larger than expected number of side effects be reported or low compliance be found. Within the first 10 participants we did not record any serious side effects. Any safety-related termination decision would be taken by the medical safety advisor.

Feedback from Public and patient involvement (PPI)- a lay interest group for clinical research

Patients and healthy lay persons interested in clinical research were invited to give feedback on the study concept and separately the specific protocol. Participants of the first PPI were positive about the possibility of a non-invasive treatment that may allow to replace medication. For the specific study protocol, PPI participants indicated that attending the TMS part with its up to 6 hours long visits for 4 days during a week would be a considerable burden for them, especially for those who suffer from pain-related partial disabilities. Other feedback criticized that this is only open for patients who are mobile, not actively working or on long term sick leave, and that a study of such dimension would need to include inconvenience allowances for participants. We hence included failure to recruit and protocol compliance in the feasibility and acceptability assessment.

Sample size

The pilot aims to recruit n= 45 (30 active, 15 sham iTBS) participants. In this pilot study, we estimate the variance of the outcome measures, and adjust for the upper confidence level (27).

Handling and storage of data

Hardcopies of consent, notes, and data will be stored securely in lockable closets. Digital data will be handled and stored safely on university-owned password-protected servers. Data will be entered in a RedCAP database. To maintain data quality one person will enter the data and a second will check

For peer review only - http://bmjopen.bmj.com/site/about/guidelines.xhtml

and validate the data. Data will be audited on a monthly basis and any issues raised at the biweekly study team meetings, one of whose functions is to monitor quality.

Data analysis

Brain imaging changes will be assessed using current version of standardised and well documented image analysis such as the NIHR Nottingham BRC imaging pipeline (https://github.com/SPMIC-UoN) that has implemented and combined routines from the most suitable advanced image analysis packages, such as FSL, MRConn and the specific protocol with statistical analysis plan will be documented before data analysis will occur.

Trial status

The study started in July 2021 until July 2024. The recruitment opened 12/05/2022 and is anticipated to be completed by 31/12/2023. As of March 2023, 28 participants completed the trial.

Given the pilot nature and small size of the trial, no DMC was needed but instead an independent experienced neurologists (RT) acted as safety advisor to optimally manage the seizure risk.

Ethics and Dissemination

All participants will provide written informed consent for taking part in the study. Ethical approval has been obtained by the South West - Cornwall & Plymouth Research Ethics Committee (REC reference:21/SW/0079). For the use of clinical facilities, approval has been obtained from Nottingham University Hospital NHS Trust (RAS 298509 Confirmation of Capacity and Capability).

The trial has been registered (Clinical Trial Reference: ISRCTN15404076, https://www.isrctn.com/ISRCTN15404076).
Results of the feasibility and pilot trial will be submitted for publication in open access, peer-review journals, for presentation at scientific conferences and may be shared with participants and PPI/E advisors.

For peer review only - http://bmjopen.bmj.com/site/about/guidelines.xhtml

Authors' contributions:

MMD performed extensive literature review of TMS pain studies and connectivity analyses, suggested part of the questionnaires, obtained ethical approval and liaised with clinical and regulatory bodies for study set up, negotiated space within suitable facilities, prepared physical setup, purchased study necessities, assisted with training, and organized feedback from lay interest groups.

DPA conceived the study, hypotheses, designed the study protocol and oversees the trial.

DH designed the CPM assessment in this study, adapted MRI scanning protocols, advised on TMS and coil positioning, takes care of day-to-day study governance and edited the manuscript during rebuttal.

SH contributes to the trial as research nurse.

BM contributed to the recruitment process design, prepared the database and paper/ electronic questionnaires and assisted with data collection.

STL provided feedback on treatment protocol.

RT contributed to the recruitment process review, safety medical review, and suggested part of the questionnaires.

SP contributed in writing the software for imaging-based personalised connectivity-guided target-selection and manuscript preparation.

CT provided support with sample size estimations, advised on the statistical design and undertakes randomization.

RM advised on the trial design and clinical outcome metrics, and provided feedback on manuscript draft stages.

DAW provided funds for the trial through his grant from Versus Arthritis UK and provided feedback on the study design and draft manuscript before submission.

MMD & DPA prepared the manuscript and edited the manuscript during rebuttal. All co-authors provided feedback on the draft before submission.

Funding statement: This trial is funded by Versus Arthritis UK and NIHR Nottingham BRC Nottingham. RT received support in part from the UK MRC (CARP MR/T024402/1). None of the funders have active roles in the design of the study, writing of the report, nor are involved in decisions regarding publications.

Competing interests statement: There are no competing interests to declare from any author.

References

1. Malfliet A, Coppieters I, Van Wilgen P, Kregel J, De Pauw R, Dolphens M, et al. Brain changes associated with cognitive and emotional factors in chronic pain: A systematic review. Eur J Pain. 2017;21(5):769-86.
2. Cottam WJ, Iwabuchi SJ, Drabek MM, Reckziegel D, Auer DP. Altered connectivity of the right anterior insula drives the pain connectome changes in chronic knee osteoarthritis. Pain. 2018;159(5):929-38.
3. Jannati A, Oberman LM, Rotenberg A, Pascual-Leone A. Assessing the mechanisms of brain plasticity by transcranial magnetic stimulation. Neuropsychopharmacol. 2023;48(1):191-208.

For peer review only - http://bmjopen.bmj.com/site/about/guidelines.xhtml

For peer review only

4. Cruccu G, Garcia-Larrea L, Hansson P, Keindl M, Lefaucheur JP, Paulus W, et al. EAN guidelines on central neurostimulation therapy in chronic pain conditions. Eur J Neurol. 2016;23(10):1489-99.
5. O'Connell NE, Marston L, Spencer S, DeSouza LH, Wands BM. Non-invasive brain stimulation techniques for chronic pain. Cochrane Db Syst Rev. 2018(3).
6. Saltychev M, Laimi K. Effectiveness of repetitive transcranial magnetic stimulation in patients with fibromyalgia: a meta-analysis. Int J Rehabil Res. 2017;40(1):11-8.
7. Yang SY, Chang MC. Effect of Repetitive Transcranial Magnetic Stimulation on Pain Management: A Systematic Narrative Review. Frontiers in Neurology. 2020;11.
8. Lefaucheur JP, Aleman A, Baeken C, Benninger DH, Brunelin J, Di Lazzaro V, et al. Evidence-based guidelines on the therapeutic use of repetitive transcranial magnetic stimulation (rTMS): An update (2014-2018). Clin Neurophysiol. 2020;131(2):474-528.
9. Galhardoni R, Correia GS, Araujo H, Yeng LT, Fernandes DT, Kaziyama HH, et al. Repetitive Transcranial Magnetic Stimulation in Chronic Pain: A Review of the Literature. Archives of physical medicine and rehabilitation. 2015;96(4):S156-S72.
10. Pszczolkowski S, Cottam WJ, Briley PM, Iwabuchi SJ, Kaylor-Hughes C, Shalabi A, et al. Connectivity-Guided Theta Burst Transcranial Magnetic Stimulation Versus Repetitive Transcranial Magnetic Stimulation for Treatment-Resistant Moderate to Severe Depression: Magnetic Resonance Imaging Protocol and SARS-CoV-2-Induced Changes for a Randomized Double-blind Controlled Trial. JMIR Res Protoc. 2022;11(1).
11. Morriss R, Webster L, Abdelghani M, Auer DP, Barber S, Bates P, et al. Connectivity guided theta burst transcranial magnetic stimulation versus repetitive transcranial magnetic stimulation for treatment-resistant moderate to severe depression: study protocol for a randomised double-blind controlled trial (BRIGhTMIND). BMJ Open. 2020;10(7).
12. Blumberger DM, Vila-Rodriguez F, Thorpe KE. Effectiveness of theta burst versus high-frequency repetitive transcranial magnetic stimulation in patients with depression (THREE-D): a randomised non-inferiority trial (vol 391, pg 1683, 2018). Lancet. 2018;391(10139):E24-E.
13. Fitzgerald PB. An update on the clinical use of repetitive transcranial magnetic stimulation in the treatment of depression. J Affect Disord. 2020;276:90-103.
14. Duprat R, Desmyter S, De Raedt R, van Heeringen K, Van den Abbeele D, Tandt H, et al. Accelerated intermittent theta burst stimulation treatment in medication resistant major depression: A fast road to remission? J Affect Disord. 2016;200:6-14.
15. Cole EJ, Stimpson KH, Bentzley BS, Gulser M, Cherian K, Tischler C, et al. Stanford Accelerated Intelligent Neuromodulation Therapy for Treatment-Resistant Depression. Am J Psychiat. 2020;177(8):716-26.
16. Millar B, McWilliams DF, Abhishek A, Akin-Akinyosoye K, Auer DP, Chapman V, et al. Investigating musculoskeletal health and wellbeing; a cohort study protocol. BMC Musculoskelet Disord. 2020;21(1).
17. Fox MD, Buckner RL, White MP, Greicius MD, Pascual-Leone A. Efficacy of Transcranial Magnetic Stimulation Targets for Depression Is Related to Intrinsic Functional Connectivity with the Subgenual Cingulate. Biol Psychiat. 2012;72(7):595-603
18. Kessler S, Grammozis A, Gunther KP, Kirschner S. The Intermittent and Constant Pain Score (ICOAP) - a Questionnaire to Assess Pain in Patients with Gonarthritis. Z Orthop Unfallchir. 2011;149(1):22-6
19. Akin-Akinyosoye K, James RJE, McWilliams DF, Millar B, das Nair R, Ferguson E, et al. The Central Aspects of Pain in the Knee (CAP-Knee) questionnaire; a mixed methods

For peer review only - http://bmjopen.bmj.com/site/about/guidelines.xhtml

study of a self-report instrument for assessing central mechanisms in people with knee pain. Osteoarthr Cartilage. 2021;29(6):802-14.
20. Freynhagen R, Baron R, Gockel U, Tolle TR. painDETECT: a new screening questionnaire to identify neuropathic components in patients with back pain. Curr Med Res Opin. 2006;22(10):1911-20.
21. Sullivan MJL, Bishop SR, Pivik J. The Pain Catastrophizing Scale: Development and validation. Psychol Assessment. 1995;7(4):524-32.
22. Williams N. The GAD-7 questionnaire. Occup Med-Oxford. 2014;64(3):224-.
23. Henry JD, Crawford JR. The short-form version of the Depression Anxiety Stress Scales (DASS-21): construct validity and normative data in a large non-clinical sample. Br J Clin Psychol. 2005;44(Pt 2):227-39.
24. Kenn C, Wood H, Kucyj M, Wattis J, Cunane J. Validation of the Hospital Anxiety and Depression Rating-Scale (Hads) in an Elderly Psychiatric Population. Int J Geriatr Phys. 1987;2(3):189-93. version 2.0 30/05/2023
25. Lowe B, Kroenke K, Herzog W, Grafe K. Measuring depression outcome with a brief self-report instrument: sensitivity to change of the Patient Health Questionnaire (PHQ-9). J Affect Disord. 2004;81(1):61-6.
26. Nasreddine ZS, Chertkow H, Phillips N, Whitehead V, Collin I, Cummings JL. The Montreal Cognitive Assessment (MoCA): a brief cognitive screening tool for detection of Mild Cognitive Impairment. Neurology. 2004;62(7):A132-A.
27. Whitehead AL, Julious SA, Cooper CL, Campbell MJ. Estimating the sample size for a pilot randomised trial to minimise the overall trial sample size for the external pilot and main trial for a continuous outcome variable. Stat Methods Med Res.2016;25(3):1057-73.

For peer review only

For peer review only - http://bmjopen.bmj.com/site/about/guidelines.xhtml

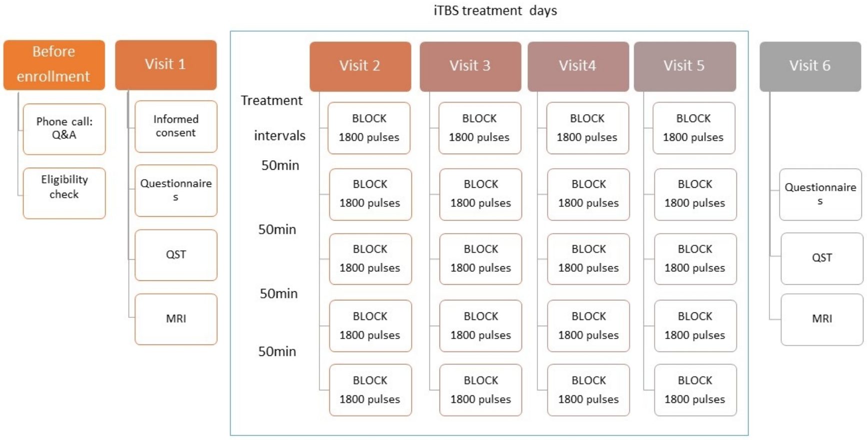

Figure 1: Pilot study overview

338x190mm (96 x 96 DPI)

STANDARD PROTOCOL ITEMS: RECOMMENDATIONS FOR INTERVENTIONAL TRIALS

SPIRIT 2013 Checklist for the ReTrain pilot RCT: Recommended items to address in a clinical trial protocol and related documents*

Section/item	Item No	Description	Addressed on page number
**Administrative information**			
Title	1	Descriptive title identifying the study design, population, interventions, and, if applicable, trial acronym	1
Trial registration	2a	Trial identifier and registry name. If not yet registered, name of intended registry	2
	2b	All items from the World Health Organization Trial Registration Data Set	See clinical trial registration
Protocol version	3	Date and version identifier	1
Funding	4	Sources and types of financial, material, and other support	15
Roles and responsibilities	5a	Names, affiliations, and roles of protocol contributors	1, 15
	5b	Name and contact information for the trial sponsor	1
	5c	Role of study sponsor and funders, if any, in study design; collection, management, analysis, and interpretation of data; writing of the report; and the decision to submit the report for publication, including whether they will have ultimate authority over any of these activities	15
	5d	Composition, roles, and responsibilities of the coordinating centre, steering committee, endpoint adjudication committee, data management team, and other individuals or groups overseeing the trial, if applicable (see Item 21a for data monitoring committee)	15,14,12,10

For peer review only

**Introduction**			
Background and rationale	6a	Description of research question and justification for undertaking the trial, including summary of relevant studies (published and unpublished) examining benefits and harms for each intervention	3- 5
	6b	Explanation for choice of comparators	10,11
Objectives	7	Specific objectives or hypotheses	6
Trial design	8	Description of trial design including type of trial (eg, parallel group, crossover, factorial, single group), allocation ratio, and framework (eg, superiority, equivalence, noninferiority, exploratory)	5
**Methods: Participants, interventions, and outcomes**			
Study setting	9	Description of study settings (eg, community clinic, academic hospital) and list of countries where data will be collected. Reference to where list of study sites can be obtained	5
Eligibility criteria	10	Inclusion and exclusion criteria for participants. If applicable, eligibility criteria for study centres and individuals who will perform the interventions (eg, surgeons, psychotherapists)	7,8
Interventions	11a	Interventions for each group with sufficient detail to allow replication, including how and when they will be administered	8-11
	11b	Criteria for discontinuing or modifying allocated interventions for a given trial participant (eg, drug dose change in response to harms, participant request, or improving/worsening disease)	12,13
	11c	Strategies to improve adherence to intervention protocols, and any procedures for monitoring adherence (eg, drug tablet return, laboratory tests)	13
	11d	Relevant concomitant care and interventions that are permitted or prohibited during the trial	11-13
Outcomes	12	Primary, secondary, and other outcomes, including the specific measurement variable (eg, systolic blood pressure), analysis metric (eg, change from baseline, final value, time to event), method of aggregation (eg, median, proportion), and time point for each outcome. Explanation of the clinical relevance of chosen efficacy and harm outcomes is strongly recommended	11-13
Participant timeline	13	Time schedule of enrolment, interventions (including any run-ins and washouts), assessments, and visits for participants. A schematic diagram is highly recommended (see Figure)	6

Sample size	14	Estimated number of participants needed to achieve study objectives and how it was determined, including clinical and statistical assumptions supporting any sample size calculations	13,14
Recruitment	15	Strategies for achieving adequate participant enrolment to reach target sample size	7
**Methods: Assignment of interventions (for controlled trials)**			
Allocation:			
Sequence generation	16a	Method of generating the allocation sequence (eg, computer-generated random numbers), and list of any factors for stratification. To reduce predictability of a random sequence, details of any planned restriction (eg, blocking) should be provided in a separate document that is unavailable to those who enrol participants or assign interventions	10
Allocation concealment mechanism	16b	Mechanism of implementing the allocation sequence (eg, central telephone; sequentially numbered, opaque, sealed envelopes), describing any steps to conceal the sequence until interventions are assigned	10
Implementation	16c	Who will generate the allocation sequence, who will enrol participants, and who will assign participants to interventions	10
Blinding (masking)	17a	Who will be blinded after assignment to interventions (eg, trial participants, care providers, outcome assessors, data analysts), and how	10
	17b	If blinded, circumstances under which unblinding is permissible, and procedure for revealing a participant's allocated intervention during the trial	10
**Methods: Data collection, management, and analysis**			
Data collection methods	18a	Plans for assessment and collection of outcome, baseline, and other trial data, including any related processes to promote data quality (eg, duplicate measurements, training of assessors) and a description of study instruments (eg, questionnaires, laboratory tests) along with their reliability and validity, if known. Reference to where data collection forms can be found, if not in the protocol	14
	18b	Plans to promote participant retention and complete follow-up, including list of any outcome data to be collected for participants who discontinue or deviate from intervention protocols	n.a.

Data management	19	Plans for data entry, coding, security, and storage, including any related processes to promote data quality (eg, double data entry; range checks for data values). Reference to where details of data management procedures can be found, if not in the protocol	10
Statistical methods	20a	Statistical methods for analysing primary and secondary outcomes. Reference to where other details of the statistical analysis plan can be found, if not in the protocol	14
	20b	Methods for any additional analyses (eg, subgroup and adjusted analyses)	n.a.
	20c	Definition of analysis population relating to protocol non-adherence (eg, as randomised analysis), and any statistical methods to handle missing data (eg, multiple imputation)	n.a.
**Methods: Monitoring**			
Data monitoring	21a	Composition of data monitoring committee (DMC); summary of its role and reporting structure; statement of whether it is independent from the sponsor and competing interests; and reference to where further details about its charter can be found, if not in the protocol. Alternatively, an explanation of why a DMC is not needed	14
	21b	Description of any interim analyses and stopping guidelines, including who will have access to these interim results and make the final decision to terminate the trial	12
Harms	22	Plans for collecting, assessing, reporting, and managing solicited and spontaneously reported adverse events and other unintended effects of trial interventions or trial conduct	13
Auditing	23	Frequency and procedures for auditing trial conduct, if any, and whether the process will be independent from investigators and the sponsor	n.a.
**Ethics and dissemination**			
Research ethics approval	24	Plans for seeking research ethics committee/institutional review board (REC/IRB) approval	1
Protocol amendments	25	Plans for communicating important protocol modifications (eg, changes to eligibility criteria, outcomes, analyses) to relevant parties (eg, investigators, REC/IRBs, trial participants, trial registries, journals, regulators)	14

For peer review only - http://bmjopen.bmj.com/site/about/guidelines.xhtml

Consent or assent	26a	Who will obtain informed consent or assent from potential trial participants or authorised surrogates, and how (see Item 32)	6
	26b	Additional consent provisions for collection and use of participant data and biological specimens in ancillary studies, if applicable	n.a.
Confidentiality	27	How personal information about potential and enrolled participants will be collected, shared, and maintained in order to protect confidentiality before, during, and after the trial	See participant information sheet
Declaration of interests	28	Financial and other competing interests for principal investigators for the overall trial and each study site	15
Access to data	29	Statement of who will have access to the final trial dataset, and disclosure of contractual agreements that limit such access for investigators	See clinical trial registry
Ancillary and post-trial care	30	Provisions, if any, for ancillary and post-trial care, and for compensation to those who suffer harm from trial participation	See participant information sheet.
Dissemination policy	31a	Plans for investigators and sponsor to communicate trial results to participants, healthcare professionals, the public, and other relevant groups (eg, via publication, reporting in results databases, or other data sharing arrangements), including any publication restrictions	14
	31b	Authorship eligibility guidelines and any intended use of professional writers	15
	31c	Plans, if any, for granting public access to the full protocol, participant-level dataset, and statistical code	n.a.
**Appendices**			
Informed consent materials	32	Model consent form and other related documentation given to participants and authorised surrogates	Attached.
Biological specimens	33	Plans for collection, laboratory evaluation, and storage of biological specimens for genetic or molecular analysis in the current trial and for future use in ancillary studies, if applicable	n.a.

*It is strongly recommended that this checklist be read in conjunction with the SPIRIT 2013 Explanation & Elaboration for important clarification on the items. Amendments to the protocol should be tracked and dated. The SPIRIT checklist is copyrighted by the SPIRIT Group under the Creative Commons “Attribution-NonCommercial-NoDerivs 3.0 Unported” license.

For peer review only
